# A Study on the Fundamental Mechanism and the Evolutionary Driving Forces behind Aerobic Fermentation in Yeast

**DOI:** 10.1371/journal.pone.0116942

**Published:** 2015-01-24

**Authors:** Arne Hagman, Jure Piškur

**Affiliations:** Department of Biology, Lund University, Lund, Sweden; Texas A&M University, UNITED STATES

## Abstract

Baker’s yeast *Saccharomyces cerevisiae* rapidly converts sugars to ethanol and carbon dioxide at both anaerobic and aerobic conditions. The later phenomenon is called Crabtree effect and has been described in two forms, long-term and short-term effect. We have previously studied under fully controlled aerobic conditions forty yeast species for their central carbon metabolism and the presence of long-term Crabtree effect. We have also studied ten steady-state yeast cultures, pulsed them with glucose, and followed the central carbon metabolism and the appearance of ethanol at dynamic conditions. In this paper we analyzed those wet laboratory data to elucidate possible mechanisms that determine the fate of glucose in different yeast species that cover approximately 250 million years of evolutionary history. We determine overflow metabolism to be the fundamental mechanism behind both long- and short-term Crabtree effect, which originated approximately 125–150 million years ago in the *Saccharomyces* lineage. The “invention” of overflow metabolism was the first step in the evolution of aerobic fermentation in yeast. It provides a general strategy to increase energy production rates, which we show is positively correlated to growth. The “invention” of overflow has also simultaneously enabled rapid glucose consumption in yeast, which is a trait that could have been selected for, to “starve” competitors in nature. We also show that glucose repression of respiration is confined mainly among *S. cerevisiae* and closely related species that diverged after the whole genome duplication event, less than 100 million years ago. Thus, glucose repression of respiration was apparently “invented” as a second step to further increase overflow and ethanol production, to inhibit growth of other microbes. The driving force behind the initial evolutionary steps was most likely competition with other microbes to faster consume and convert sugar into biomass, in niches that were semi-anaerobic.

## Introduction

One of the most prominent features of the baker’s yeast *Saccharomyces cerevisiae* is the rapid conversion of sugars to ethanol and carbon dioxide at both anaerobic and aerobic conditions. When oxygen is absent, acetaldehyde is the final electron acceptor and gets converted into ethanol under purely fermentative growth. Under aerobic conditions, respiration is possible with oxygen as the final electron acceptor, but *S. cerevisiae* still exhibits alcoholic fermentation until the sugar/glucose is depleted from the medium. This phenomenon is called the Crabtree effect [1]. However, it is possible to obtain pure respiratory growth under aerobic conditions if the glucose concentration is kept very low in the medium, e.g. by using glucose-limited continuous culture operating below a certain strain-specific threshold value (called “critical” dilution rate) or by using fed-batch cultivations [2]. Briefly, the yeast cell senses glucose, and this signal is transmitted further to diminish the respiratory activities. This glucose repression phenomenon involves different signal transduction pathways activated by extracellular and intracellular levels of glucose and related metabolites and/or fluxes through the key glycolytic enzymes [3]. Some of the key regulatory genes are: *GLC7, REG1, HXK2, SNF1*, and *MIG1, MIG2, MIG3*, as well as several other involved in the glucose sensing pathway (*RGT1, RGT2* and *SNF3*) and also several genes to be first identified in the future. In other words, a complexity of glucose repression regulatory networks is still far to be completely understood. Some of the regulatory activities operate at the transcription regulation level and some may operate directly on the involved enzymes and their regulators. While glucose sensing and its regulatory mechanisms are relatively well understood in *S. cerevisiae* [4–6], they have been less studied in other yeast species. A crucial question is if the glucose repression of the respiratory pathway is the only cause of alcoholic fermentation and if it was also the first step during the evolution of this phenomenon.

Different physiological and molecular approaches have been used as the background for the current definitions of Crabtree effect [7–10]. In this study we follow the generally accepted definitions and describe the long-term Crabtree effect as aerobic alcoholic fermentation under steady-state conditions at high growth rates [7]. When *S. cerevisiae* is cultivated in a glucose-limited chemostat, the long-term effect appears when the dilution rate (or the glucose uptake rate) exceeds the strain specific threshold value. In other words, when the specific growth rate in chemostat exceeds the so-called “critical specific growth rate” (determined by the critical dilution rate), ethanol starts accumulating. The same effect is observed also when yeast cells are cultivated at high glucose conditions, e.g. batch cultivations [2, 11]. The underlying mechanism behind this physiology is not yet fully understood, but believed to be caused by repression of genes involved in respiration. On the other hand, we define, as basically proposed by Pronk and colleagues [12] the short-term Crabtree effect as the immediate appearance of aerobic alcoholic fermentation upon addition of excess sugar to sugar-limited and purely respiratory cultures. This effect has also been explained as an overflow in the sugar metabolism, initially caused by physiological constraints that could be associated directly with the biochemical properties, such as maximum velocity and feed-back regulation, of the respiration-associated enzymes and their regulators [2, 12, 13]. In addition, it could depend on immediate repression of some key genes involved in respiration. It is still unclear if the regulatory molecular mechanisms operating during the long-term and short-term Crabtree effect are indeed different, and a very interesting aspect is the evolutionary background and the origin of the Crabtree effect [14].

Until recently very few yeast species have been systematically studied for their carbon metabolism [15]. Studies on different yeasts with a clear phylogenetic relationship can, among other aspects, also help to elucidate the evolutionary history of yeast carbon metabolism, as well as they can shed light on the basic mechanisms behind the observed traits. In our recent work we have studied over forty different Saccharomycetales yeasts for the presence of long-term Crabtree effect and it has been found that this effect originated after the divergence of the *Saccharomyces-Lachancea* and *Kluyveromyces-Eremothecium* lineages, prior to the whole genome duplication (WGD) event and after the loss of the respiratory complex I [16]. We have also cultivated ten of those yeast species under steady-state conditions, and with glucose as the limiting factor [17]. Upon glucose pulse, we have studied the consumption of glucose and formation of ethanol, and other fermentation and respiration products. We have demonstrated that both effects, short-term and long-term Crabtree effect originated at approximately the same time during the Saccharomycetales evolutionary history [16, 17], and the origin occurred with the horizontal transfer of *URA1* gene [18] and the ability to proliferate anaerobically [15, 19], after the loss of respiratory complex I [20, 21].

In this paper, we analyzed those wet experimental results we had obtained in our previous studies on short-term [17] and long-term Crabtree effect [16] to elucidate possible mechanisms, which determine the fate of glucose in different yeast species. All short- and long-term Crabtree positive yeasts are hereafter designated as respiro-fermenting yeasts, and all short- and long-term Crabtree negative yeasts are designated as purely respiring yeasts (Table 1). We interpret our results as that overflow is likely the fundamental and primary mechanism behind both short- and long-term Crabtree effect, and that the origin of overflow in sugar metabolism precedes the origin of glucose repression of respiration. We also elaborate on the driving force underlying overflow mechanism and the evolution of aerobic fermentation in yeasts.

**Table 1:** Investigated species and their designation.

**Species**	**Y**	**Designation**	**LCP**	**SCP**	**GRR**
*Sac. p. Weihenstephan*	Y1288	RF	+	nd	+
*Sac. cerevisiae*	Y706	RF	+	+	+
*Sac. paradoxus*	Y052	RF	+	nd	+
*Sac. mikatae*	Y393	RF	+	nd	nd
*Sac. uvarum*	Y1124	RF	+	nd	+
*Sac. eubayanus*	Y1693	RF	+	nd	+
*Kaz. lodderae*	Y489	RF	+	nd	nd
*Kaz. exiguus*	Y670	RF	+	nd	+
*Kaz. barnettii*	Y477	RF	+	nd	+
*Nau. castellii*	Y056	RF	+	nd	nd
*Nak. glabrata*	Y475	RF	+	nd	nd
*Nak. delphensis*	Y476	RF	+	nd	nd
*Nak. castellii*	Y484	RF	+	nd	nd
*Tet. phaffii*	Y482	RF	+	nd	nd
*Tet. iriomotensis*	Y1299	RF	+	nd	nd
*Van. polysporus*	Y1293	RF	+	+	nd
*Van. yarrowii*	Y1677	RF	+	nd	nd
*Zto. florentinus*	Y479	RF	+	nd	nd
*Zto. mrakii*	Y480	RF	+	nd	nd
*Tor. franciscae*	Y1055	RF	+	+	nd
*Lac. fermentati*	Y083	RF	+	nd	nd
*Lac. thermotolerans*	Y688	RF	+	nd	nd
*Lac. waltii*	Y1062	RF	+	+	nd
*Lac. kluyverii*	Y057	RF	+	+	nd
*Klu. aestuarii*	Y797	R	-	nd	nd
*Klu. nonfermentans*	Y1057	R	-	nd	nd
*Klu. wickerhamii*	Y113	R	-	nd	nd
*Klu. lactis*	Y707	RF	-	+	nd
*Klu. marxianus*	Y1674	R	-	nd	nd
*Klu. marxianus*	Y1675	R	-	nd	nd
*Klu. marxianus*	Y1058	R	-	-	nd
*Klu. dobzhanskii*	Y796	RF	+	nd	nd
*Ere. coryli*	Y999	R	-	-	nd
*Ere. sinecaudum*	Y1002	R	-	nd	nd
*Deb. vanrijiae*	Y060	R	-	-	nd
*Pic. philogaea*	Y074	R	-	nd	nd
*Pic. pastoris*	Y1294	R	-	-	nd

LCP = long-term Crabtree positive — data from [16]

SCP = short-term Crabtree positive — data from [17]

GRR = glucose repression of respiration

RF = respiro-fermenting yeast

R = respiring yeast

nd = not determined

## Results and Discussion

### Respiro-fermenting yeasts possess a constantly switched on fermentative pathway

When yeasts proliferate on glucose, a flux of carbon will occur through the central carbon metabolism. The flux of carbon through any pathway depends on enzymatic activities, and therefore on the presence of enzymes belonging to this particular pathway. The activities of pathways can be quantified by measuring the formation rates of metabolic products or consumption rates of substrates. Carbon flux in anabolic pathways can end up as biomass, and in catabolic pathways it can result in either formation of CO_2_ from complete oxidation (respiration) or a mixture of CO_2_ and metabolites, such as ethanol, from incomplete oxidation (anaerobic glycolysis). Since rapid ethanol formation can be observed in all short-term Crabtree positive yeasts (S1 Fig. and Table 2), it can be concluded that at least these yeast species possess a significant abundance of all the enzymes that constitute the pathway for anaerobic glycolysis, even under glucose limited and aerobic growth conditions. In other words, even when these yeasts “starve”, their capacity to convert glucose to ethanol is switched on.

**Table 2:** Yeast short-term Crabtree effect — Growth kinetics for all experiments.

**Species**	**Y**	**Time: minutes**	Cons. rate*: Glucose	Prod. rate*: Ethanol	Growth rate*: DW	Prod. rate*: CO_2_	Cons. Rate**: O_2_	**RQ**
*Sac. cerevisiae B*	Y706	SS	6,7	0,0	0,1	2,8	2,8	1,0
		5–20	16,9	5,4	2,3	6,7	3,8	1,8
		60–150	32,2	12,3	7,6	7,7	2,8	2,8
*Sac. cerevisiae A*	Y706	SS	6,3	0,0	0,1	2,6	2,6	1,0
		5–20	22,0	5,3	1,4	7,2	3,9	1,9
		60–150	29,8	10,9	5,6	7,2	3,3	2,1
*Van. polysporus A*	Y1293	SS	6,8	0,0	0,1	2,8	2,7	1,0
		5–20	46,4	8,2	3,7	8,9	4,2	2,1
		60–130	38,4	9,7	6,8	9,7	3,5	2,7
*Tor. franciscae B*	Y1055	SS	4,9	0,0	0,1	2,3	1,2	1,9
		5–20	11,4	3,0	8,5	4,5	2,5	1,8
		60–150	24,3	6,8	4,2	6,3	3,2	2,0
*Tor. franciscae A*	Y1055	SS	4,6	0,0	0,1	2,1	1,5	1,4
		5–20	12,0	2,6	5,0	4,3	2,4	1,8
		60–150	23,5	6,9	4,0	6,0	3,0	2,0
*Lac. waltii B*	Y1062	SS	10,3	0,0	0,1	Nd	Nd	Nd
		5–20	22,2	5,1	14,6	Nd	Nd	Nd
		60–180	18,1	4,7	5,7	Nd	Nd	Nd
*Lac. waltii A*	Y1062	SS	9,9	0,0	0,1	2,6	3,0	0,9
		5–20	18,9	4,5	11,2	5,5	3,5	1,6
		60–180	18,0	4,7	5,6	5,5	3,4	1,6
*Lac. kluyverii B*	Y1651	SS	7,7	0,0	0,1	2,9	2,9	1,0
		5–20	16,1	3,2	-4,5	5,7	4,0	1,4
		60–170	25,1	9,1	7,4	7,4	4,1	1,8
*Lac. kluyverii A*	Y1651	SS	7,9	0,0	0,1	3,2	3,2	1,0
		5–20	20,2	2,7	1,8	5,6	4,4	1,3
		60–170	25,1	8,5	7,9	8,0	4,6	1,7
*Klu. lactis B*	Y707	SS	6,1	0,0	0,1	0,9	0,9	1,0
		5–20	14,6	0,1	6,9	5,2	4,5	1,2
		60–210	16,9	1,9	6,3	5,7	4,9	1,2
*Klu. lactis A*	Y707	SS	6,0	0,0	0,1	0,9	1,0	1,0
		5–20	17,3	1,6	6,4	4,8	4,4	1,1
		60–210	16,5	2,5	6,5	5,0	4,6	1,1
*Klu. marxianus B*	Y1058	SS	6,5	0,0	0,1	2,5	2,1	1,2
		5–20	14,8	-1,1	8,1	1,5	1,5	1,0
		60–210	12,1	0,0	8,1	1,4	1,3	1,1
*Klu. marxianus A*	Y1058	SS	6,3	0,0	0,1	3,0	2,2	1,3
		5–20	14,4	0,9	9,8	1,5	1,6	1,0
		60–210	11,9	0,0	8,1	1,4	1,4	1,0
*Ere. coryli B*	Y999	SS	4,3	0,0	0,1	0,7	0,8	0,9
		5–20	1,2	0,6	4,4	0,7	0,7	1,1
		60–735	2,3	-0,1	1,1	0,5	0,5	1,1
*Ere. coryli A*	Y999	SS	3,9	0,0	0,1	0,7	0,8	1,0
		5–20	4,3	1,2	5,3	0,8	0,8	1,1
		60–735	1,9	-0,2	1,4	0,6	0,5	1,1
*Deb. vanrijiae B*	Y060	SS	5,0	0,0	0,1	1,9	2,0	0,9
		5–25	9,7	0,0	1,7	2,2	2,3	1,0
		70–260	8,3	0,0	6,2	2,3	2,2	1,1
*Deb. vanrijiae A*	Y060	SS	5,0	0,0	0,1	1,7	2,1	0,8
		5–25	7,2	-0,7	4,0	2,3	2,3	1,0
		70–260	8,4	0,0	6,0	2,3	2,0	1,1
*Pic. pastoris B*	Y1294	SS	4,4	0,0	0,1	1,5	1,4	1,1
		5–20	14,2	0,0	13,9	2,4	2,1	1,2
		80–320	6,7	0,0	6,0	1,7	1,4	1,2
*Pic. pastoris A*	Y1294	SS	4,3	0,0	0,1	1,5	1,3	1,2
		5–20	17,3	0,0	20,2	2,4	2,0	1,2
		80–320	6,9	0,0	5,1	1,9	1,6	1,1

Data derived from [17]

* C-mmole/gDW*h

** mmole/gDW*h

Nd = Not determined

SS = Steady-state

### Respiro-fermenting yeasts possess a high capacity of energy production even at low growth rates

**Fig 1:**
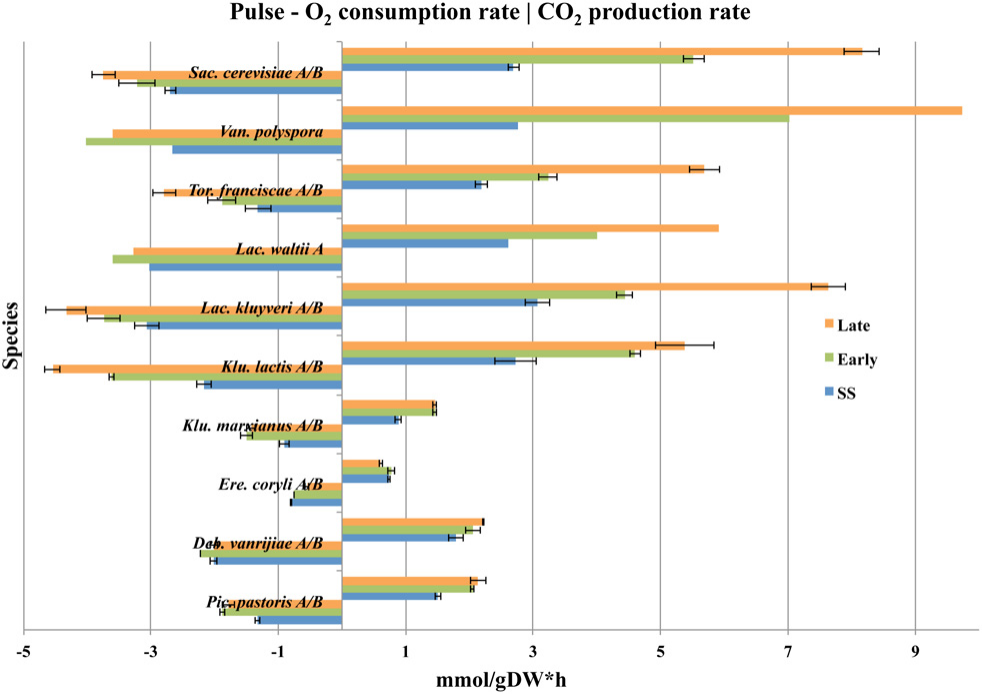
Yeast O_2_ consumption and CO_2_ production rates. ATP is produced primarily from respiration, but also from fermentation. When yeast respires, O_2_ will be consumed. CO_2_ is produced from both respiration as well as fermentation. This figure illustrates that all short-term Crabtree positive yeasts have an initially higher energy metabolism that increases rapidly as compared to short-term Crabtree negative yeasts, as the steady-state cultures leave glucose limited growth and enter higher growth-rates in excess of glucose. The average O_2_ consumption (left) and CO_2_ production rates (right) between replicates are illustrated for cultures at steady-state (SS), time interval 5–20 minutes (Early-phase), and time interval after 60 minutes (Late-phase) while glucose was still present after a glucose-pulse. Error bars correspond to the standard deviation.

**Fig 2:**
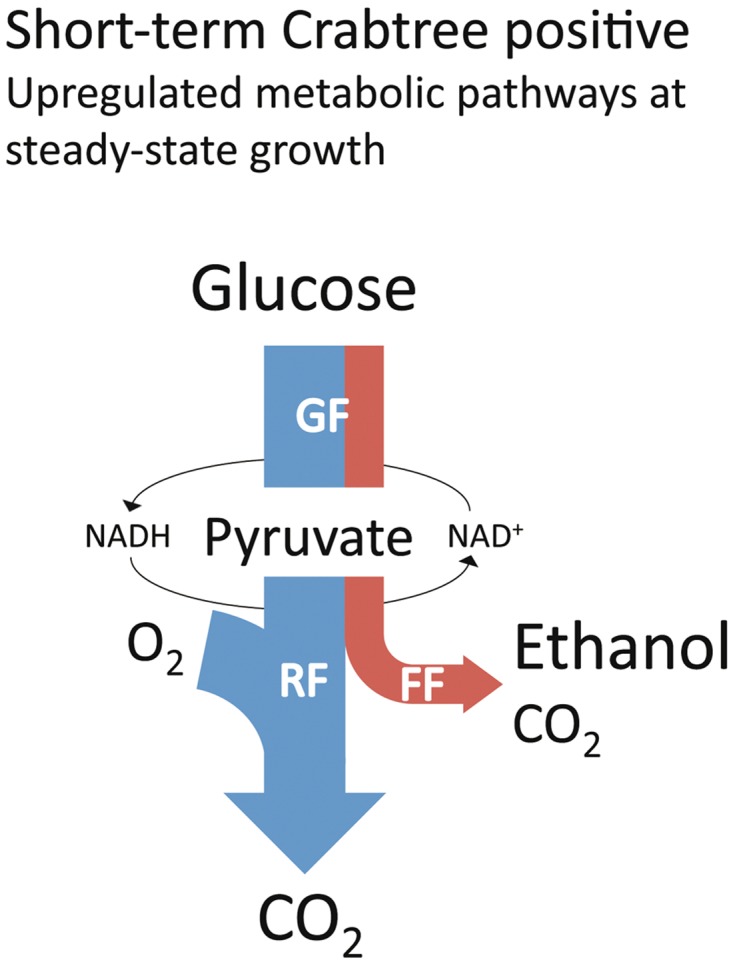
Yeast central carbon metabolic pathways at steady-state growth. All short-term Crabtree positive yeasts possess an upregulated aerobic (blue) and anaerobic (red) glycolytic pathway, even under fully aerobic conditions, when energy and carbon-source is limiting. At low glucose uptake rates, yeasts cells are purely respiring and there is a carbon-flux only through glycolysis (GF) and respiration (RF). Upon a sudden glucose excess condition (glucose pulse), the glycolytic flux will exceed the respiratory flux, which results in a fermentative flux (FF) and ethanol production. In other words, it appears as if slowly dividing short-term Crabtree positive cells, with little food around are already equipped with a strong energy producing apparatus, for rapid glucose consumption and energy production.

Growing cells need ATP as an energy source to fuel the assembly of biomolecules, and drive the flux of carbon in anabolic pathways in a cell. Respiration is the most energy efficient pathway, which accounts for the production of almost 8 times more ATP as compared to anaerobic glycolysis. Respiration is therefore the most powerful pathway for maintaining a high ATP/ADP ratio in growing cells under aerobic conditions. This activity can be estimated from the consumption rates of O_2_. Anaerobic glycolysis is on the other hand an energy-producing pathway that can function without O_2_, which makes it the important pathway for maintaining the ATP/ADP ratio under anaerobic conditions only. The activity of anaerobic glycolysis can be estimated from the production rates of CO_2_, since this pathway produces CO_2_ without any consumption of O_2_. Thus, a positive correlation between energy metabolism and growth is expected. We have investigated the catabolic activities in short-term Crabtree positive and Crabtree negative yeasts, by comparing their O_2_ consumption and CO_2_ production rates (Fig. 1 and Table 2). It is clear that short-term Crabtree positive yeasts started consuming O_2_ and producing CO_2_ at high rates immediately after a glucose pulse, as compared to the Crabtree negative yeasts. These results demonstrate that even when short-term Crabtree positive yeasts “starve”, they maintained an upregulated energy-producing apparatus, which was expressed as initially higher respiration and fermentation rates per gram of biomass as compared to the Crabtree negative yeasts (Fig. 2).

### Uncoupled upregulation of anaerobic glycolysis and respiration in respiro-fermenting yeasts

**Fig 3:**
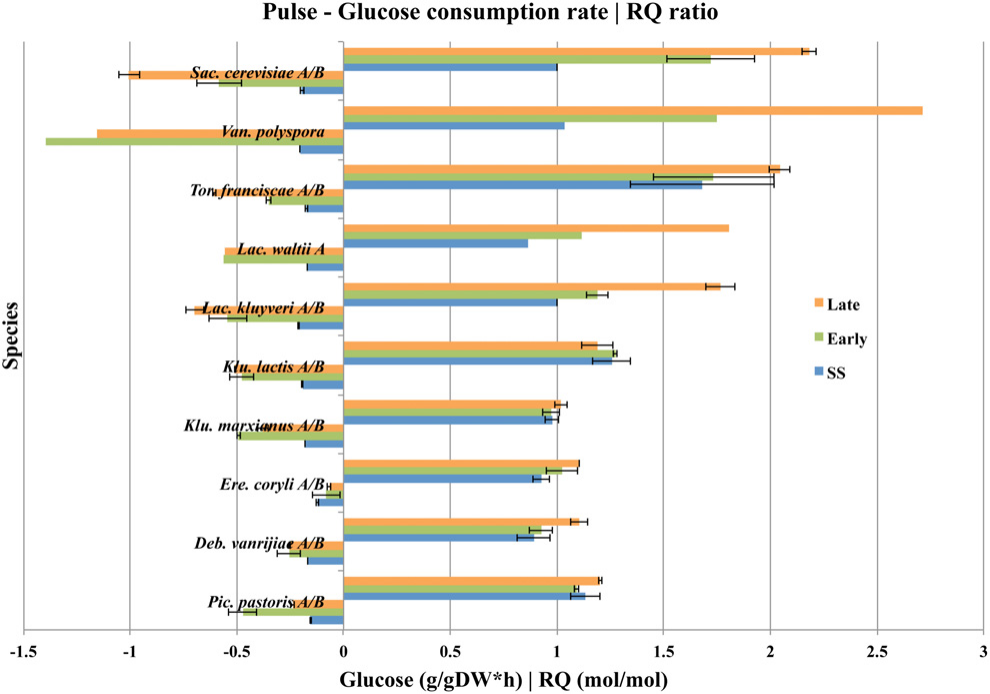
Yeast glucose consumption rate and respiratory quotient (RQ). Under purely respiratory metabolism at low biomass formation (growth) rates, roughly one CO_2_ molecule will be produced for each O_2_ molecule consumed. Fermentation does not require molecular O_2_ as the final electron acceptor, and will only produce one CO_2_ in the first decarboxylation step of each pyruvate. Hence, RQ ratios that are significantly greater than one indicate a fermentative activity. All ethanol-forming yeasts have RQ ratios significantly greater than one, while all non-ethanol forming yeasts have an RQ close to, or equal to one. The average glucose consumption rates (left) and RQ ratios (right) between biological replicates are illustrated for cultures at steady-state (SS), time interval 5–20 minutes (Early-phase) and time interval after 60 minutes (Late-phase) while glucose was still present after a glucose pulse. Error bars correspond to the standard deviation.

All short-term Crabtree positive yeasts also successively increased their energy producing apparatus in the presence of glucose, which was mainly accomplished by the upregulation of both fermentative and glycolytic pathways. This is further illustrated with respiratory quotients (RQ), which is calculated as the ratio between fermentative and respiratory activities (Fig. 3 and Table 2). We show that all short-term Crabtree positive yeasts (except *K. lactis*) exhibited high initial fermentative activities as compared to respiration, at early time intervals (first 20 minutes) after a glucose pulse. The fermentative activities increased during the adaptation phase to higher growth rates at later time intervals (more than 60 minutes), after the release from glucose limiting growth conditions. Since no significant decrease in O_2_ consumption rates could be observed in any of the short-term Crabtree positive yeasts after a glucose-pulse (compare with Fig. 1), the increase in respiratory quotient can only be a result from an upregulation of anaerobic glycolysis. This is also indicated by increased glucose uptake rates. In the case of *K. lactis*, it can be seen that its RQ did not change significantly upon a glucose pulse with values just above 1.1, which is consistent with a weak fermentative activity that was not much higher than the Crabtree negative yeasts.

### Short-term Crabtree effect may be triggered by uncoordinated regulation between glucose uptake and growth

We have demonstrated that respiro-fermenting yeasts already possessed an upregulated fermentative pathway (S1 Fig.), and a large energy producing apparatus (Figs. 1 and 2), even under aerobic and glucose limiting growth conditions. We have also shown that uncoupled regulation of the glycolytic and respiratory pathways could be responsible for the increased flux through fermentative pathways, which is expressed as short-term Crabtree effect in all respiro-fermenting yeasts (Fig. 3). When a pulse of glucose was applied to these yeasts, an increase in carbon flux through the glycolytic and fermentative pathways relative to the respiratory pathways was observed. This suggests that separate regulatory networks govern anaerobic glycolysis and respiration in short-term Crabtree positive yeasts.

**Fig 4:**
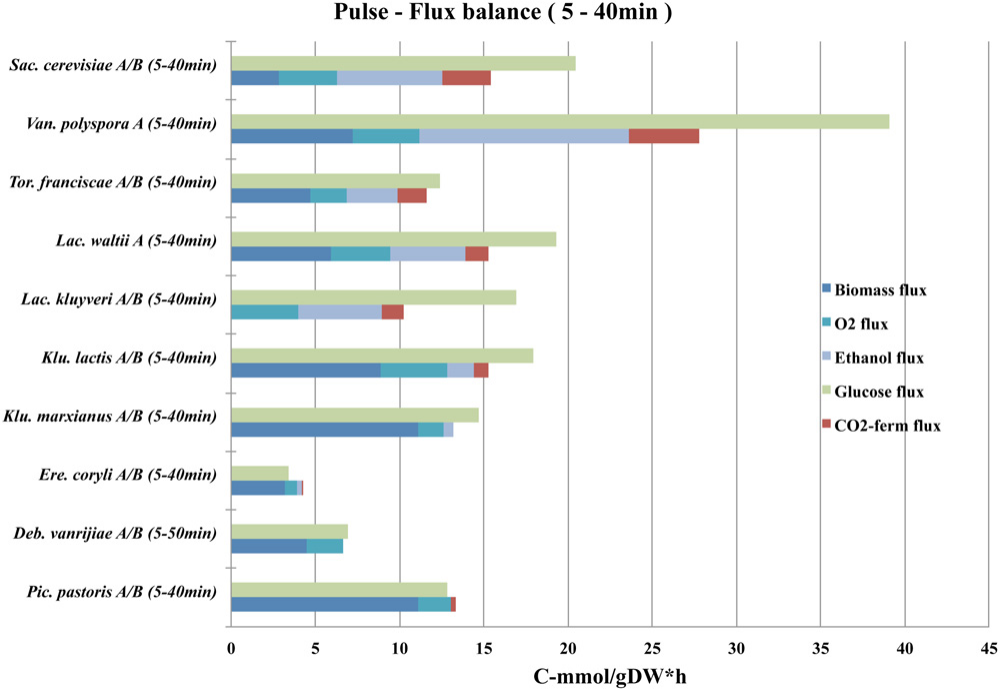
Carbon-flux balance. Glucose was the main carbon and energy source for cell proliferation in all experiments. The sugar is taken up by cells, and metabolized to form biomolecules and release energy for growth. The flow of carbon in central carbon metabolism was estimated from the average formation rates in a time interval of end products such as, biomass, CO_2_ from fermentation and respiration, and ethanol. These output rates should be lower than or roughly equal to the input rates of carbon into a cell, which is equivalent to the average glucose uptake rates. As expected, glucose uptake rates were high for short-term Crabtree positive yeasts, which is a necessity to maintain high carbon-flux through fermentative pathways. It is clear that fermenting yeasts possessed a high glycolytic flux and at least a basal activity of enzymes that constitute the fermentative pathway. It is also clear that *K. lactis*, which had a glycolytic capacity close to intermediate fermenting yeast, such as *L. kluyverii, L. waltii* and *T. franciscae* only fermented weakly, and thus appeared to have a retarded response to glucose. This can however be explained by the higher anabolic flux in *K. lactis*, which directs the carbon flow from fermentative pathways to biomass formation.

To further investigate for any differences in the regulation of energy-associated metabolism and growth in short-term Crabtree positive and Crabtree negative yeasts, we investigated the differences in carbon flux through anabolic and catabolic pathways for all yeast species. This was accomplished with a carbon flux-balance, where average rates at certain time intervals for glucose consumption were compared to the average rates of carbon dioxide production (from both respiration and fermentation), ethanol production, and biomass production (Fig. 4 and Table 2). It can be shown that the initial growth rates were highly unstable during the transition from glucose limited steady-state growth to glucose excess growth in all yeasts (see also S8 Fig.). Interestingly, our flux-balance also reveals higher glucose consumption rates as compared to biomass formation and respiration in all short-term Crabtree positive yeasts. This short-term imbalance between anabolic and catabolic pathways was not observed in any of the Crabtree negative yeasts, for any of the investigated time intervals. Thus, high glucose uptake rates (that exceeded biomass formation and respiration) lead to unbalanced carbon-flux between glycolysis and anabolic pathways, what resulted in increased flux through fermentative pathways and ethanol formation in short-term Crabtree positive yeasts.

Our carbon-flux analysis (Fig. 4) suggests that an uncoupled regulation of glycolysis from biomass formation (and respiration) could be the mechanism behind short-term Crabtree effect. Can the degree of unbalanced metabolism also explain the different degrees of short-term Crabtree effect in the fermenting yeast species? Our results reveal that the level of short-term ethanol production is not only positively affected by smaller biomass flux (i.e. *K. lactis* as compared to *L. kluyverii*) but also by greater glycolytic flux that exceeds both the biomass and respiratory flux (i.e. *K. lactis* as compared to *V. polyspora*). These observations suggest that short-term Crabtree positive yeasts must possess a strong glucose uptake apparatus, even at glucose limiting conditions and at low growth rates. It appears as if glucose uptake is not strictly synchronized with growth and can therefore easily exceed the anabolic activity, which could be the underlying factor that triggered the observed short-term Crabtree effect in *K. lactis*, *L. kluyverii, L. waltii, T. franciscae, V. polyspora* and *S. cerevisiae*. These yeasts immediately take up glucose upon a glucose pulse, which is then catabolized to pyruvate. Because growth and respiration represents bottlenecks, pyruvate enters the available fermentative pathway.

### The origin of short-term Crabtree effect precedes repression of respiration

A fundamental trait that distinguishes *S. cerevisiae* from Crabtree negative yeasts is its ability to repress respiration as a long-term response to glucose at high growth rates, such as in batch cultures. Since long-term glucose pulse experiments could in theory be considered as equivalent to batch cultivation, a comparison between early and late time intervals after a glucose pulse should be able to reveal any long-term responses. Our results show no such long-term repression of respiration in any of the investigated short-term Crabtree positive yeasts for the first 20 minutes as compared to time intervals after 60 minutes (Fig. 1). However, a new type of long-term glucose effect can be observed in all short-term Crabtree positive yeasts (except *K. lactis*) that appear to affect the upregulation of the anaerobic glycolytic pathway only (compare with Fig. 3). These yeasts exhibit increasing RQ-values at later time intervals after a pulse of glucose.

**Fig 5:**
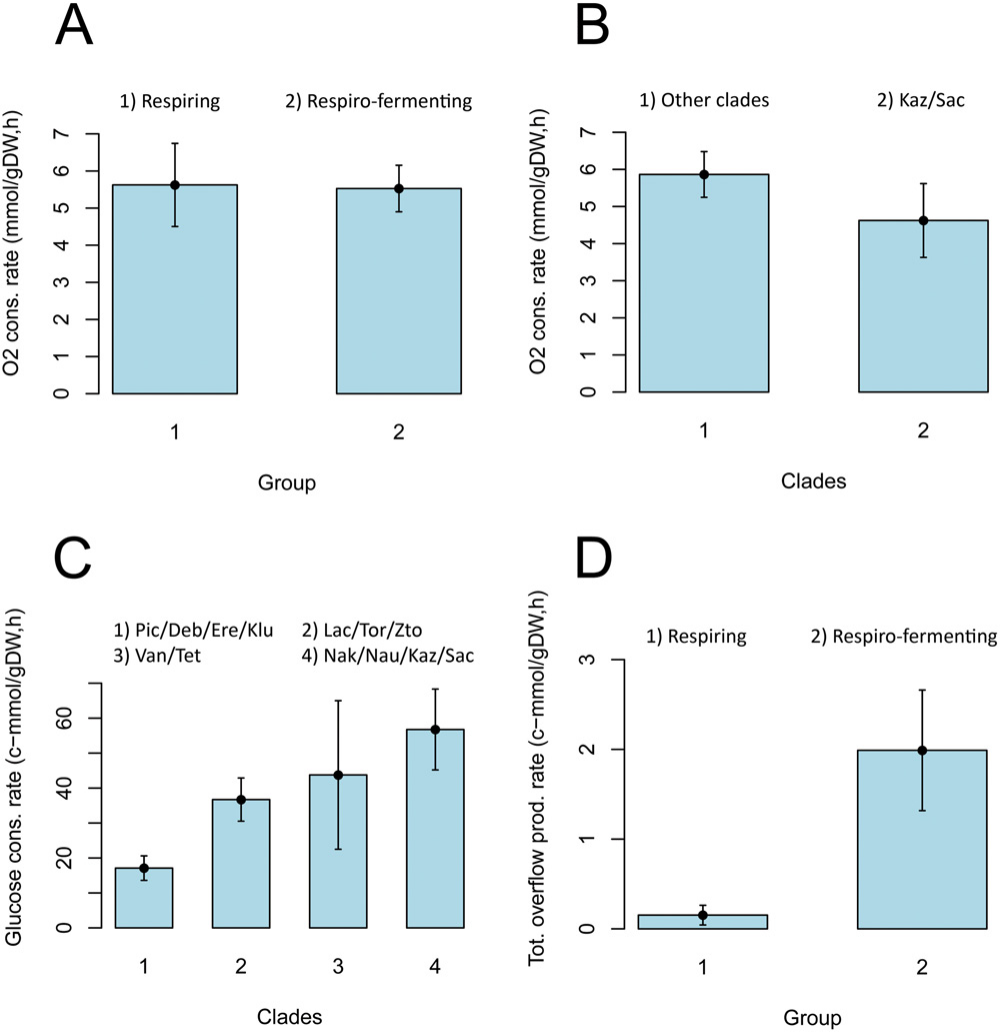
The origin of overflow precedes the origin of glucose repression of respiration. These figures illustrate the difference in population means among different phylogenetic groups and parameters (see Table 1 for designations) with error bars corresponding to the 95% confidence interval. **(A)** No significant difference in O_2_ consumption rates could be observed between purely respiring yeasts and respiro-fermenting yeasts. **(B)** Among all of the respiro-fermenting yeasts, only members of the *Kazachstania* and *Saccharomyces* clades appear to possess repression of respiration. **(C)** This figure is adapted from an earlier study [16], and illustrates how glucose consumption rates have evolved with the gradual increase of ethanol fermentation in the *Saccharomyces* lineage. Purely respiring yeasts constitute group 1 and respiro-fermenting yeasts constitute groups 2, 3 and 4 as previously defined [16]. **(D)** Overflow metabolites other than ethanol, such as acetate, pyruvate, glycerol, lactate and succinate were readily detected in all respiro-fermenting yeasts as compared to purely respiring yeasts. Thus, the origin of aerobic fermentation coincides with the origin of overflow metabolism in the *Saccharomyces* lineage.

**Fig 6:**
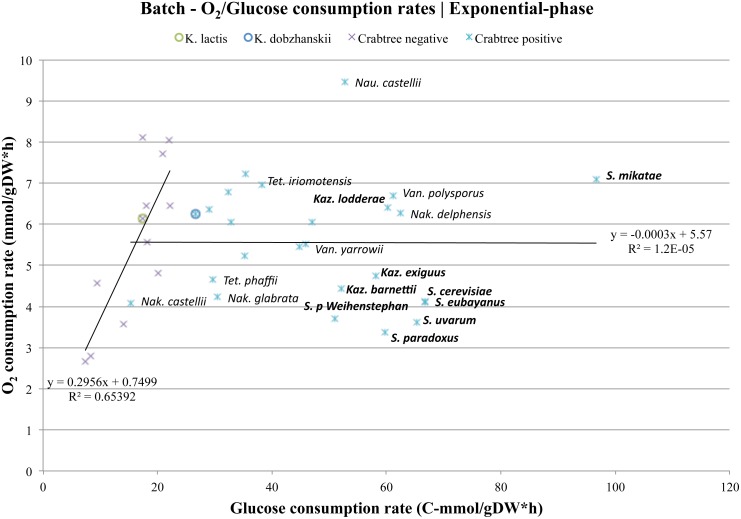
Long-term glucose repression of respiration. Oxygen and glucose consumption rates were determined from batch cultures of over forty yeast species at their exponential growth phase [16]. It is known from studies on *S. cerevisiae* that it exhibits repressed respiration when cultivated at high growth-rates on glucose, even under aerobic batch conditions. This trait was named after it’s discoverer H.G Crabtree [1] and was originally associated with glucose repression of respiration in the mammal cell. Our results confirm the early observations made by de Deken for *S. cerevisiae* and sister-species, but surprisingly most of the other respiro-fermenting yeast species appear to lack this trait. Although our results can confirm the existence of repression of respiration in a majority of yeast species that belong to the *Saccharomyces* and *Kazachstania* clade (see also Fig. 5B and S10D Fig.) we cannot rule out the possibility that this peculiar trait might occur in any of the other early branching clades, nor the existence of several regulatory pathways that could govern glucose repression.

Could it be that glucose repression of respiration is strictly associated with gene-regulation at high growth-rates? When we compared the O_2_ consumption rates in forty different yeast species from batch cultures (S1 Table), no significant differences between respiro-fermenting and purely respiring yeasts could be detected (Fig. 5A and Table 3). Hence, no long-term repression of respiration could be detected in at least the majority of respiro-fermenting yeasts, and the origin of aerobic fermentation does not coincide with the origin of glucose repression of respiration. Could it then be that glucose repression of respiration is fully expressed only in *S. cerevisiae* and its WGD sister species? Indeed, when we compared the O_2_ consumption rates in *S. cerevisiae* and its closest relatives with all other yeasts, significantly lower O_2_ consumption rates could be detected only among the members of the *Saccharomyces* and *Kazachstania* clades (Figs. 5B and 6, Table 3). These results suggest that the origin of strong glucose repression of respiration occurred relatively late in the yeast evolutionary history, likely after the whole genome duplication event (WGD). Thus, the origin of glucose repression of respiration and Crabtree effect, as we know it in *S. cerevisiae*, was preceded by the origin of aerobic fermentation and short-term Crabtree effect. On the other hand, long-term glucose activation of the anaerobic glycolytic pathway appears to have evolved just before the divergence of the *Lachancea* and *Saccharomyces* lineages (see also Fig. 5C and S2 Fig.). This mechanism has to our knowledge never been demonstrated before.

**Table 3:** Statististical tests on metabolic groups.

**Parameter**	**Group 1**	**Group 2**	**Statistical analysis**	**α (5%)**	**p-value**	**df**
Sum of overflow production rates*	Respiring	Respiro-fermenting	Welch Two Sample t-test	Yes	4.8E-06	24.1
			Wilcoxon rank sum test	Yes	2.5E-08	
Oxygen consumption rates	Respiring	Respiro-fermenting	Welch Two Sample t-test	No	8.7E-01	20.5
			Wilcoxon rank sum test	No	7.4E-01	
Oxygen consumption rates	Other clades	*Saccharomyces/ Kazachstania*	Welch Two Sample t-test	Yes	3.1E-02	16.5
			Wilcoxon rank sum test	Yes	4.4E-02	

*Sum of acetate, pyruvate, glycerol, succinate and lactate was accessed

### Overflow is the mechanism behind short- and long-term responses

**Fig 7:**
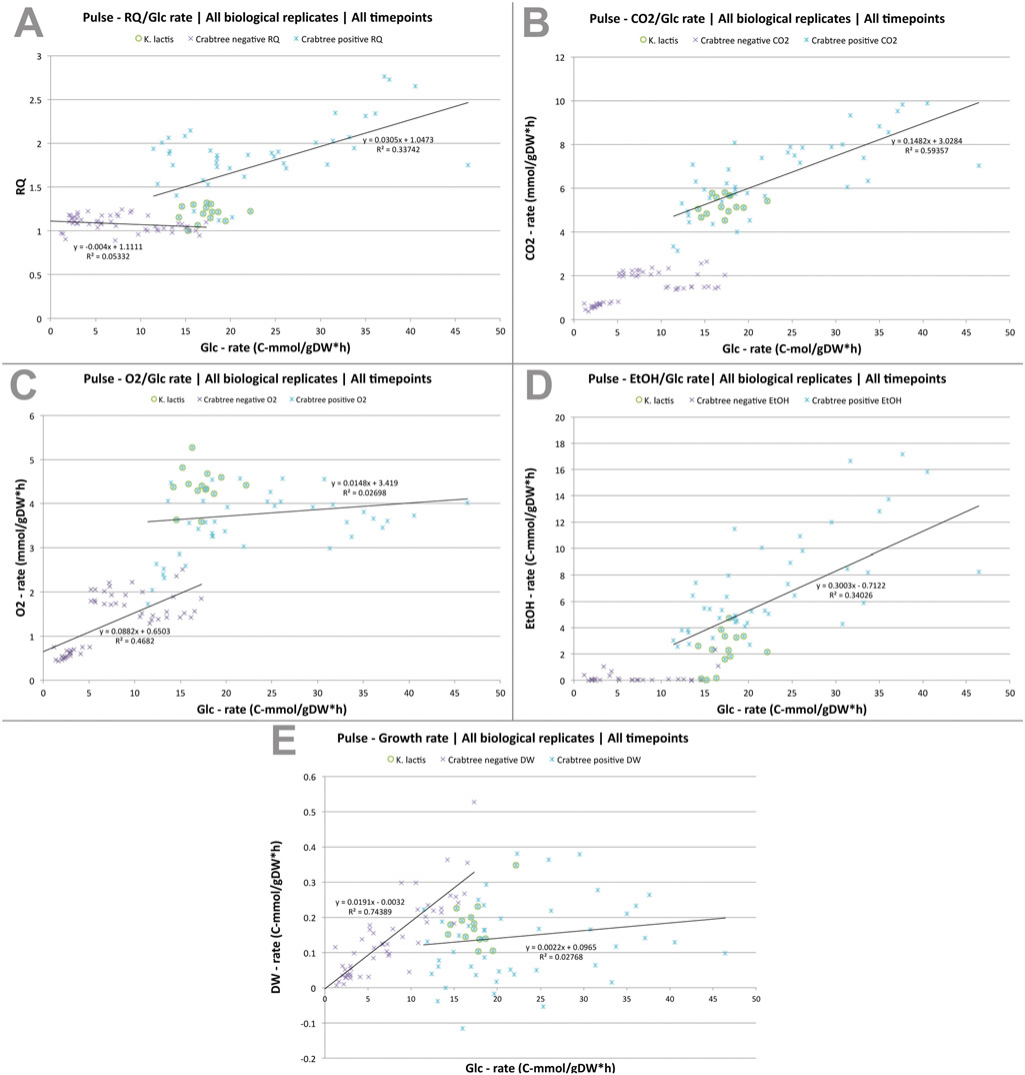
Pulse—Growth parameters in correlation with glucose uptake rates at early and late growth phases. The average glucose uptake rates for all time intervals and investigated species [17] are plotted against the average of (**A**) RQ, (**B**) CO_2_ production rates, (**C**) O_2_ consumption rates, (**D**) ethanol production rates, and (**E**) growth rates at the corresponding time intervals, while glucose is still present after a glucose-pulse. Yeast species can be grouped according to their glucose uptake rates and fermenting capacity. The critical glucose uptake rate (GF_crit_) is defined as the rate where overflow occurs, what separates fermenting from non-fermenting yeasts. There is an interrelationship between fermentative and respiratory pathways that depend on glucose uptake rates among the investigated species. A linear correlation between ethanol fermentation and glucose uptake rates can be deduced from the data (starting at GF_crit_). The linear correlations are highly variable at early time intervals (see also S3 Fig.), but become more apparent at later time intervals, when the cultures were more adapted to the new growth conditions (see also S4 Fig.).

We have shown that a balance exist between glucose uptake rates and carbon-flux through anabolic and catabolic pathways in short-term Crabtree negative yeast species (Fig. 4). Uncoordinated glucose uptake with growth causes imbalance between the catabolic and anabolic pathways that result in overflow of respiration, which further leads to increased carbon-flux through fermentative pathways in short-term Crabtree positive yeasts. The interrelationship between pathways is apparent in short-term responses to glucose, and is greatly affected by biomass formation rates. This can be observed in weak short-term Crabtree positive yeasts such as *K. lactis*, due to its initially lower glucose uptake rates. We have also shown that repression of respiration appears to occur only as a long-term response to glucose at high growth-rates, such as in batch cultures of *S. cerevisiae* and closely related species (Fig. 5B). Glucose repression of respiration is therefore not a major contributor to the observed imbalanced flow of carbon through the glycolytic and other anabolic and catabolic pathways in the majority of short-term Crabtree positive yeasts. In order to investigate for any evolutionary conserved interrelationship between fermentative and respiratory pathways among species, we expanded our analysis to interspecies comparison of (I) glucose uptake rate and (II) RQ, ethanol production rate, CO_2_ production rate, O_2_ consumption rate, and growth rate for all species and time intervals (Fig. 7 and Table 2). From these comparisons, several conclusions can be drawn; (I) yeast species can be grouped as respiro-fermenting and purely respiring based on their glucose uptake rates, (II) a universal cut-off value for glucose uptake rate appears around 10–15 C-mmol/gDW*h for all investigated yeast species (III) although the values are highly variable (see further below), an uniform and linear relationship between glycolysis and fermentation can be observed among species above the cut-off value and, (IV) *K. lactis* is at the border-line above the cut-off value for glucose uptake rate, just before overflow occurs.

It can also be concluded that much of the variance in the data set was caused by an unstable growth in the transition from growth under glucose limiting- to glucose excess- conditions, which was primarily observed among respiro-fermenting yeast species within the first hour (S3 Fig. and Table 2). These conclusions are more apparent, when early time intervals are compared with later ones. At late time intervals, more coherent trends can be observed when the cultures were more adapted to new glucose rich conditions and growth sets in, also for respiro-fermenting yeasts (S4 Fig. and Table 2).

### A universal model for overflow metabolism in yeast

Overflow metabolism seem to be the fundamental mechanism behind short-term Crabtree effect in all investigated yeast species, and a delayed regulation of cell-growth as compared to an intrinsically upregulated anaerobic glycolysis, could transiently shift long-term Crabtree negative yeasts to short-term Crabtree positive yeasts, such as in the case of *K. lactis*. Furthermore, overflow also appears to play an important role at later stages after a glucose pulse, and since long-term glucose pulse experiments are in principal equivalent to batch-cultivations, similar conclusions could be valid for both approaches. These results, together with the fact that the origin of short-term Crabtree effect coincides with the origin of long-term Crabtree effect [17], further suggests overflow to be the mechanism behind both traits. To test this hypothesis, similar analyses were done on a dataset from a large-scale batch study of forty different yeast species [16], and the results are clear (Fig. 6 and S5 Fig., S1 Table). Indeed the cut-off value for glucose uptake rate before overflow occurs is approximately 15 C-mmol/gDW*h (determined as described under the Materials and Methods section). In these figures *K. lactis* is highlighted together with *K. dobzhanskii*, and it can be seen that the former is at the borderline of the cut-off value for overflow, but this time grouped together with Crabtree negative yeast species, while *K. dobzhanskii* is not.

Our analysis reveals an universal and linear interrelationship between the fermentative and glycolytic activities among species, with a set-off value at the critical-glucose uptake rate (GF_crit_) at approximately 15 C-mmol/gDW*h, following the model:

$$\begin{array}{cc} \text{GF}=\text{BF}+\text{RF}+{\text{k}}^{*}\text{FF}\;\; & \text{k}=1\;\text{for}\;\text{GF}>{\text{GF}}_{\text{crit}}\\ & \text{k}=0\;\text{for}\;\text{GF}\leq {\text{GF}}_{\text{crit}} \end{array}$$ (1)

In this model, GF correspond to Glycolytic Flux (estimated with glucose uptake rate), BF corresponds to Biomass Flux (estimated with growth rate), RF corresponds to Respiratory Flux (estimated with oxygen consumption rate) and FF corresponds to Fermentative Flux (estimated as the overflow flux leading to ethanol formation). It can be shown that GF_crit_ = BF + RF, and with the average biomass formation rate of 9.4 C-mmol/gDW*h and the average maximal oxygen consumption rate of 5.2 C-mmol/gDW*h for all yeasts, this would correspond to an average glucose uptake rate of 15 C-mmol/gDW*h. Simplified, when the glucose uptake rate reaches a certain rate, overflow in sugar metabolism occurs and results in the production of ethanol.

Another way to illustrate this is to correct the total glycolytic flux for biomass and respiratory flux for each species according to; GF — BF — RF = k*FF and to plot these corrected values against the corresponding ethanol production rates. The result reveals an almost perfect linear correlation between these parameters (S6A Fig.).

### An alternative model for overflow metabolism in yeast

It is also possible to introduce an additional parameter TOF into the model, which stand for Total Overflow Flux and is the sum of the formation of all other detected overflow metabolites (acetate, glycerol, succinate, pyruvate and lactate). Thus, the modified model for total overflow becomes:

$$\begin{array}{cc} \text{GF}=\text{BF}+\text{RF}+{\text{k}}^{*}\text{FF}+\text{TOF}\;\; & \text{k}=1\;\text{for}\;\text{GF}>{\text{GF}}_{\text{crit}}\\ & \text{k}=0\;\text{for}\;\text{GF}\leq {\text{GF}}_{\text{crit}} \end{array}$$ (2)

However, the introduction of a further parameter does not improve the model much, what can be illustrated by further correcting the total glycolytic flux for biomass, respiratory and total overflow flux for each species according to; GF — BF — RF — TOF = k*FF and again to plot these values against the corresponding ethanol production rates (S7A Fig.). The difference between the models is further illustrated by balancing out the carbon flows, and since the sum of several overflow products (TOF) is not significant as compared to the total flux, the addition of the variable TOF to model (1) does not improve it significantly under the applied growth conditions (S6B and S7B Figs.).

### Several putative bottlenecks behind overflow metabolism

We have previously shown that TCA-cycle intermediates such as succinate, the end product of glycolysis such as pyruvate, and intermediates in the pyruvate dehydrogenase bypass such as acetate are readily detected during growth in a majority of respiro-fermenting yeast species [16]. Here we show that the sum of all accumulated overflow metabolites are highly significant in respiro-fermenting yeasts, and absent in purely respiring yeasts (Fig. 5D and S1 Table). This suggests that there are several potential bottlenecks for overflow to occur that might reside at pyruvate or beyond. An example of one such potential bottleneck is the consumption of TCA-cycle intermediates (i.e. succinate) for amino acid production and cell-growth, upon a glucose pulse to steady-state cultures at low glucose limited growth (Fig. 4). Another example is the observed short-term intrinsic limitation in oxygen consumption rates among respiro-fermenting yeasts (Fig. 7C), and the long-term limitation in oxygen consumption rates among both respiro-fermenting and purely respiring yeast species (Fig. 6). Hence, our results suggest an universal maximal capacity of respiratory flux that can support a maximal capacity of biomass flux in our growth conditions for all investigated yeast species, with glycolytic flux as the important variable that determines overflow (equation 1 and 2 above).

### Overconsumption is a competitive strategy to maximize energy output rates

Our data and the linearity of our model predict a metabolic flux-control as the most likely fundamental mechanism to sense glucose and control its uptake [22], even if the onset of fermentation is due to overflow. It is known that respiro-fermenting yeasts possess many genes encoding low-affinity glucose transporters, which facilitate glucose uptake in an energy independent manner when glucose concentration is high. It is also known that some long-term Crabtree negative yeast species such as *K. lactis* also possess low affinity glucose transporters [23, 24], while other Crabtree negative yeasts depend on energy-dependent high-affinity transporters, for glucose uptake at low concentrations [25, 26]. An uncontrolled and relaxed glucose uptake and glycolysis that is not tightly coupled to respiration and growth, could explain the difference in short-term Crabtree effect between i.e. *K. lactis* and other Crabtree negative yeasts such as *K. marxianus*, and why the former can initially exceed GF_crit_, but not the latter.

**Fig 8:**
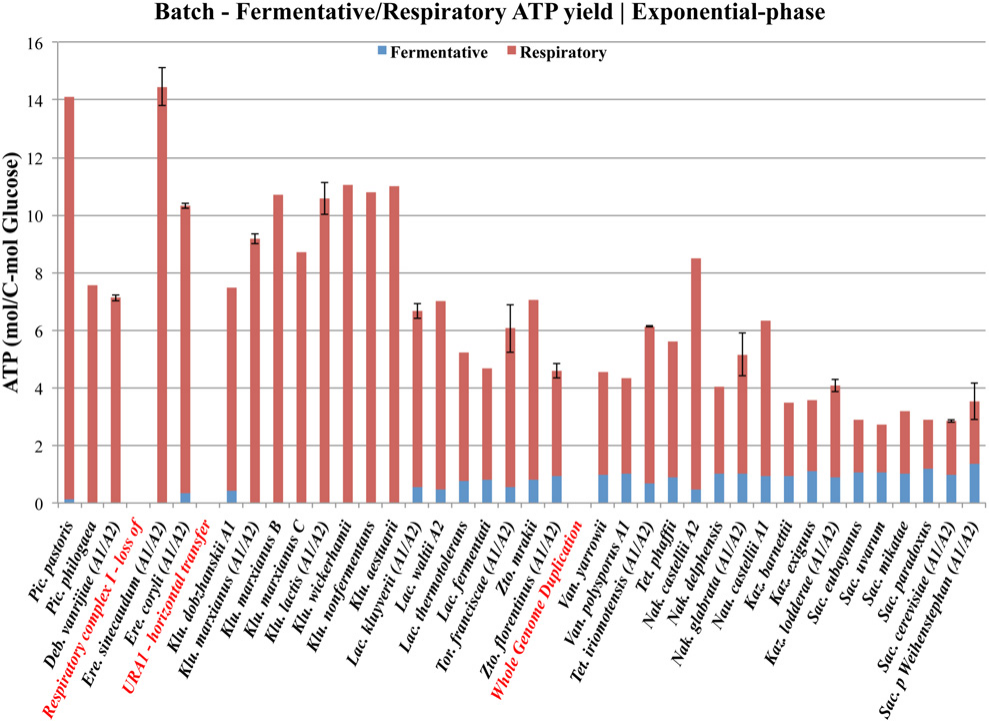
Evolution of long-term Crabtree effect in the *Saccharomyces-*lineage. This figure illustrates an overview of the evolution of long-term Crabtree effect, what resulted in lower energy-yield in *Saccharomyces* yeast species that possess the respiro-fermentative lifestyle. Theoretical ATP yields from anaerobic glycolysis (in blue) and respiration (in red) with standard deviations from two biological replicates were calculated during exponential growth-phase, using already published data [16]. Yeast species are ordered along the horizontal plane, roughly according to their reported phylogenetic relationship [29]. The timing of several evolutionary events that are relevant for the modern traits, such as the loss of respiratory complex I, the horizontal transfer of *URA1*, and the whole genome duplication (WGD) event are highlighted in red. The evolution of the peculiar trait of Crabtree positive yeast species, which appears less energy-efficient as compared to their Crabtree negative counterpart have been discussed from an ecological aspect and explained by the “make-accumulate-consume” strategy [14].

Another very interesting aspect of aerobic fermentation is the evolutionary background for the development of these regulatory mechanisms. Recently several hypotheses have emerged mainly from *in silico* approaches, and many of the evolutionary events that have been determined from comparative genomics approaches have previously been verified by comparative physiology approaches and discussed in the evolutionary context [16]. The long-term Crabtree effect has hitherto been limited to few reference species only, and has often been quantified from yields of metabolites and biomass. Hence, it is not strange that the long-term Crabtree effect has appeared, from an energetically point of view, as a very peculiar trait (illustrated in Fig. 8). Nevertheless, the evolutionary background for the development of aerobic fermentation has since the discovery of the budding yeast and its biological activities by Louis Pasteur, more than a century ago, remained unsolved.

**Fig 9:**
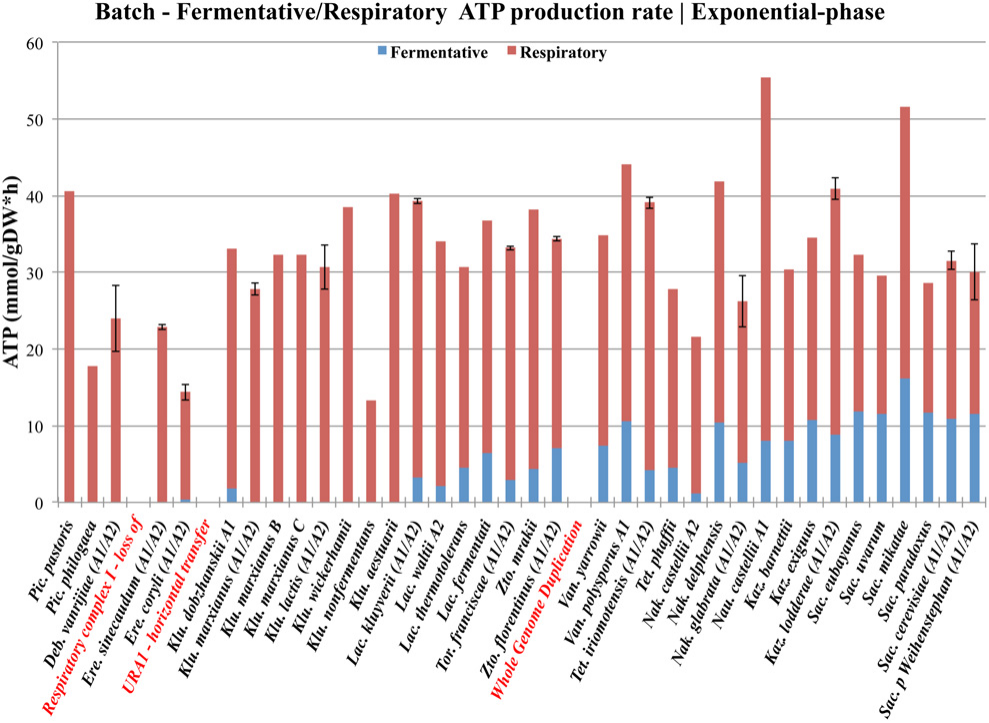
Distribution of theoretical ATP production rates in yeast. This figure illustrates an overview of the evolution of the theoretical ATP production rates from anaerobic glycolysis (in blue) and respiration (in red) in the *Saccharomyces* lineage. While the evolution of Crabtree effect has resulted in lower energy-yield in *Saccharomyces* yeast species that possess the respiro-fermentative lifestyle, the sum of ATP production rates remain fairly unchanged between the different groups of yeasts. If phylogeny is not taken into account, a positive correlation between overflow metabolism and ATP production rates can be observed (see also S9 Fig.). ATP production rates with standard deviations from two biological replicates were calculated during exponential growth-phase, using already published data [16]. Yeast species are ordered along the horizontal plane, roughly according to their phylogenetic relationship [29]. The timing of several evolutionary events that are relevant for the modern traits, such as the loss of respiratory complex I, the horizontal transfer of *URA1* and the whole genome duplication (WGD) event, are highlighted in red.

The conclusions from our study revealed overflow as the fundamental mechanism behind both short- and long-term Crabtree effect in all investigated yeast species (summarized by equation 1 and 2 above). We speculate that the fermentative lifestyle originally evolved as an advantageous trait in a readily increasing glucose-rich environment that coincides with the appearance of the first angiosperms in nature [16]. This “selfish” trait represents an efficient depletion of the most energy-rich carbon sources in short time, to maximize the energy output in a time-dependent manner for anabolic reactions and for cell proliferation (Fig. 9). The strategy to increase anaerobic glycolysis even under aerobic conditions, for increased energy output rates is even more apparent when phylogeny is not taken into account (S9 and S10A-C Figs.). Thus, it appears as if the “invention” of overflow metabolism most likely provided the ancient yeast with a competitive advantage that enabled rapid glucose consumption, primarily for growth, but also to starve competitors.

### Fine-tuning of evolutionary conserved pathways to promote overflow

Only within the members of the *Saccharomyces* and *Kazachstania* clades can a trade-off between energy and ethanol production be detected (S10D Fig.). This is of course a consequence of lower oxygen consumption rates among these yeasts (see also Fig. 5B). The strategy of fine-tuning the interrelationship between evolutionary conserved pathways, such as anaerobic glycolysis and respiration are highly compatible and flexible, since both pathways are energy producing (Fig. 9). An increased anaerobic glycolytic activity would also enable recycling of NADH for glycolysis to proceed and simultaneously drive anabolic reactions by directing the overflow of carbon to less energy-rich metabolites such as ethanol (Fig. 2). One way to increase overflow would of course require the uncoupled regulation of respiration from glycolysis in respiro-fermenting yeasts [27], so that anaerobic glycolysis can be independently upregulated, even under aerobic condition. In other words, glycolytic flux would be less tightly regulated with respiration and biomass formation. Another way to accomplish imbalance between respiration and glycolysis could also be from the gross duplication of glycolytic genes, what has been observed in WGD yeasts [28]. However, it should be noted that the origin of short-term Crabtree effect [17], and the long-term upregulation of anaerobic glycolysis predates the WGD event (S2 Fig.).

### The evolution of an alternative use of overflow metabolism

**Fig 10:**
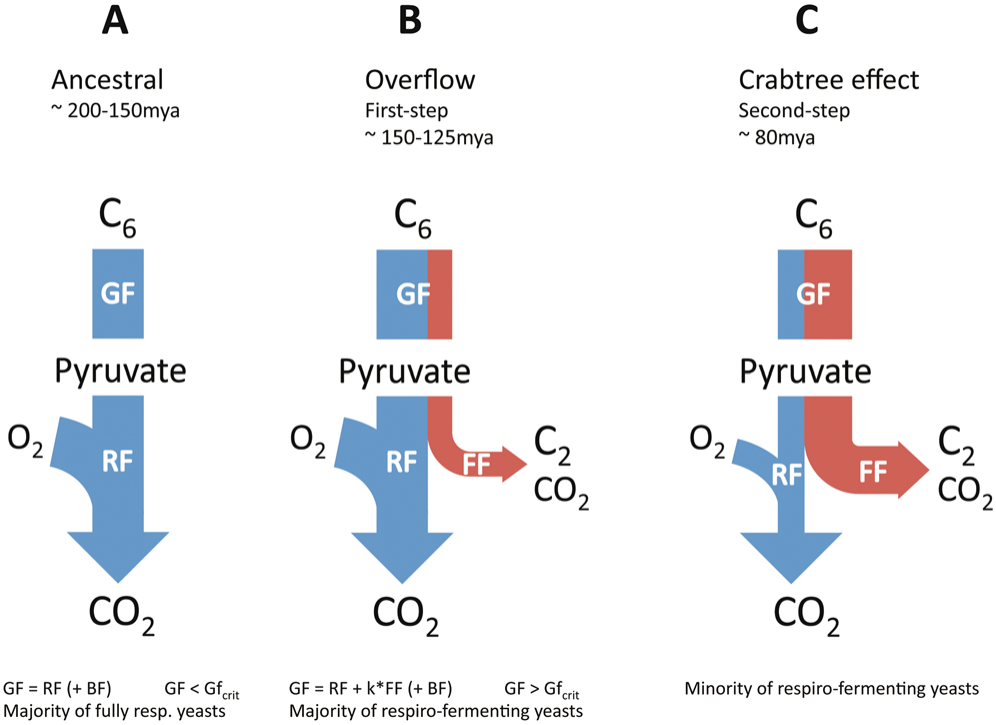
Evolutionary scenario for the origin of Crabtree effect in *Saccharomycetales* yeast. This figure illustrates the capacity of central carbon metabolic pathways for the metabolic groups of yeast (as designated in Table 1), when grown on C_6_-sugars such as glucose. Biomass formation rates have been left out, since no significant differences amongst groups could be observed (S2 Table). **(A)** Purely respiring yeasts, including *Pichia*, *Debaromyces, Eremothecium* and a majority of *Kluyveromyces* exhibited low glycolytic flux (GF), without any overflow metabolism (see also S10B Fig.). **(B)** Yeast that separated from the *Eremothecium* lineage, including some *Kluyveromyces*, and all *Lachancea, Torulaspora, Zygotorulaspora* and the majority of WGD yeasts possessed a greater glycolytic flux than respiratory flux (RF) capacity, what results in overflow metabolism. The upregulation of the anaerobic glycolysis has provided this group of yeast with a greater energy producing apparatus that can consume glucose more rapidly under aerobic conditions (see also S10C Fig.). **(C)** Our results can be interpreted as that traits such as overconsumption of glucose, and excess of energy producing capacity has enabled the development of a third metabolic group (including a majority of *Kazachstania* and *Saccharomyces*) that exhibit a trade-off between ethanol and energy production efficiency (see also S10D Fig.).

At growth-conditions that are rich enough in free sugars to support a high biomass content, other limiting factors will set in, i.e. oxygen and nitrogen depletion. It is under these semi-anaerobic conditions that overflow metabolites, such as ethanol can be accumulated in sufficient amount that they become toxic. The winning traits under these conditions would then be the ability to efficiently consume glucose through overflow, to cope with anaerobiosis such as the uncoupling of important biosynthetic pathways from respiration, and to increase resistance toward accumulation of toxic intermediates. Since respiro-fermenting yeasts share these traits, it could be assumed that these yeasts would also benefit from using ethanol as a weapon to outcompete other microbes by evolving additional mechanisms, such as repression of respiration to increase the ethanol yields further (Fig. 10). This trade-off between reduced ATP and increased ethanol production is weak, but was significant enough to be detected under the applied experimental conditions (S10D Fig.). Our data suggests that the “invention” of glucose repression of respiration occurred relatively late among the WGD yeasts, and became a settled trait with the separation of the *Saccharomyces* and *Kazachstania* clades (Fig. 5B and S2 Fig.). Hence, it appears as if modern yeast species such as *S. cerevisiae* might have found additional uses of an ancient innovation, which involves conversion of acetaldehyde to ethanol to inhibit growth of other microbes under oxygen-limited conditions, but more recently also under aerobic conditions. However, the competitive advantages of glucose repression of respiration remain to be proven in future competition studies under controlled conditions, where it can be fully expressed and quantified. It is also clear that our data on the complex trait of Crabtree effect and aerobic fermentation need to be complemented by other aspects, such as the mechanisms behind glucose uptake, long-term upregulation of anaerobic glycolysis, and the existence of glucose repression of respiration in various yeast species that diverged prior to and after the whole genome duplication event.

## Materials and Methods

### Yeast strains and growth conditions

All species that were analyzed in this study roughly cover the *Saccharomycetales* and are and listed in supplementary Table 2 and S1 Table. Furthermore, all data were derived from cultivations that were conducted under controlled and aerobic conditions, and with glucose as the only limiting carbon and energy source. More detailed information on the experimental procedures that have generated the data can be found elsewhere [16, 17].

### Estimation of the average critical glucose uptake rate among species

The critical glucose uptake rate (GF_crit_) was calculated as the intersection between the linear model for respiro-fermenting yeasts and purely respiring yeasts, for (I) RQ values (II) O_2_ consumption rates (III) ethanol production rates and (IV) growth rates. For CO_2_ production rates, GF_crit_ was calculated as the intersection between the linear model and the average O_2_ consumption rates for respiro-fermenting yeasts (Fig. 5 and S5 Fig.). According to equation (1), the average GF_crit_ can also be estimated as the sum of the average biomass flux (in C-mmol/gDW*h) and the average respiratory flux (in mmol/gDW*h).

### Theoretical ATP production rates

The theoretical ATP production rates from anaerobic glycolysis can be calculated from the ethanol production rates. Likewise, the theoretical ATP production rates from respiration can be calculated from the O_2_ consumption rates. It is estimated that the production of 1 mole of ethanol yield 1 mole of ATP, and the consumption of 1 mole of O_2_ yield 5 mole of ATP. Since the production of acetate and glycerol are not significant, the ATP production and consumption in the formation of these compounds can be neglected.

### Statistical analysis

Statistical tests and correlation analyses were performed under R (2.15.2). Parametric and non-parametric pairwise tests (Welch two sample t-test and Wilcoxon rank sum test) were applied on group-means for the total sum of overflow production and O_2_ consumption rates, which is illustrated in Fig. 5 and summarized in Table 3. We further investigated the contribution of several growth-kinetics parameters to the total sum of ATP production and growth rates with correlation analysis, which is illustrated and summarized in supplementary S10 Fig., Supplementary Material online. More specific information on the analysis and the applied statistical tests can be found in the attached (S1 Script).

## Supporting Information

S1 Fig Pulse—Glucose uptake and ethanol production rates. All short-term Crabtree positive yeasts already possess an upregulated anaerobic glycolytic pathway under aerobic, glucose limited, and fully respiring steady-state growth at low rates (SS). This was expressed as an immediate ethanol formation within 20 minutes (Early-phase) after a glucose pulse in all the fermenting yeasts. Several of the fermenting yeast species such as, *S. cerevisiae, T. franciscae* and *L. kluyverii* also significantly increased their glucose uptake rates, and ethanol production rates at time intervals later than 60 minutes (Late-phase) after a glucose pulse. The average glucose uptake and ethanol production rates from two biological replicates with error bars corresponding to the standard deviation are illustrated.
(TIF)

S2 Fig Phylogenetic distribution of Crabtree effect among investigated yeasts. This figure illustrate the distribution of short-term Crabtree effect and long-term Crabtree effect in all the investigated yeasts from our previous studies [16, 17]. Yeast species that are short-term Crabtree positive (framed in red), short-term Crabtree negative (framed in blue), long-term Crabtree positive (highlighted in red), and long-term Crabtree negative (highlighted in blue) are shown. Several evolutionary events that are relevant for the modern traits are also shown. Some events that have left a clear fingerprint in the modern genomes (whole arrows), such as the rewiring of RGE (rapid growth elements) [27], the whole genome duplication event [30], the horizontal transfer of *URA1* [18], and the loss of respiratory complex I [20], have been more precisely timed, while the timing of complex traits (broken arrows), such as overflow metabolism, long-term upregulation of anaerobic glycolysis and glucose repression of respiration (in bold), petite positivity and the capability of anaerobic growth [15], might be less precise. Figure adapted from [29].
(TIF)

S3 Fig Pulse—Growth parameters in correlation with glucose uptake rates at early growth phase. Similar analysis as in Fig. 7, but only for early time intervals, up to 20 minutes after a glucose-pulse. The data is highly variable due to low and unstable growth, and cultures had yet to adapt to the new glucose-rich environment (see also S4 Fig.).
(TIF)

S4 Fig Pulse—Growth parameters in correlation with glucose uptake rates at late growth phase. This figure illustrate a similar analysis as in Fig. 7, but only for time intervals later than 60 minutes after a glucose-pulse, when the cultures were more adapted to the new growth conditions. The data is less variable as compared to earlier time intervals (S3 Fig.).
(TIF)

S5 Fig Batch—Growth parameters in correlation with glucose uptake during exponential growth phase. Similar analysis as in Fig. 7, but the data is from a study of forty different yeast species from batch cultures [16]. Under these conditions, the cultures are fully adapted to high glucose levels and are growing at their specific growth rates. The dataset is highly correlated and it is therefore possible to determine more precisely the average GF_crit_ among species to 15 C-mmole/gDW*h (see Materials and Methods section for further information).
(TIF)

S6 Fig Evolutionary conserved metabolic flux-control among yeasts. When glucose uptake rates exceed the sum of carbon flow through biomass formation and respiration, an overflow through anaerobic glycolysis is observed, which is predicted by model (1). (**A**) An interrelationship between evolutionary conserved metabolic pathways, which occurs in a linear fashion that spans at least the evolutionary history of over forty investigated yeast species (and perhaps as far back as to the formation of dikarya-clade or even further). The slope (k), which can be derived from the plot equals 1, and correspond to the relationship between fermentative flux (FF) and glycolytic flux (GF) above the critical glucose uptake rate (GF_crit_), according to model (1). (**B**) C-flux balancing was used to validate model (1), which is illustrated in this figure.
(TIF)

S7 Fig Improved model for evolutionary conserved metabolic flux-control. Similar analysis was made as illustrated in S6A and B Fig., but with an additional variable in model (2), in an attempt to improve the model. Some variation in the dataset is caused by the formation of other overflow products, which is not accounted for in model (1). This model is already highly linearly correlated with an R^2^ value of 96, so the incorporation of an extra variable only improves the model slightly to an R^2^ value of 97 (compare S7B Fig. with S6B Fig.).
(TIF)

S8 Fig Pulse—Carbon-flux balance. This figures illustrate a carbon-flux balance similar to Fig. 4, but for the time intervals **(A)** 5 to 20 minutes and **(B)** 20 to 40 minutes. Apart from the observations and conclusions that can be drawn in Fig. 4, this figure further illustrates the unstable growth that sets in upon a sudden release from glucose limiting growth. The average of two biological replicates with the corresponding standard deviation is illustrated. Data was obtained from [17].
(TIF)

S9 Fig Overflow metabolism can contribute to increased ATP production rates. When the sum of theoretical ATP production rates from anaerobic glycolysis and respiration are plotted against glucose consumption rates, a general trend can be observed. Increased glucose consumption rates can result in higher ATP production rates. Hence, overflow metabolism enables increased ATP production rates for cell-proliferation on glucose. This figure also shows that *S. cerevisiae* and a majority of its closely related species, in the *Saccharomyces* and *Kazachstania* clades, appear to lack increased ATP production rates, despite high glycolytic flux. This can be explained by glucose repression of respiration, which is a trait that appears to have evolved late in modern yeasts. Glucose repression of respiration could have provided other competitive advantages in glucose rich conditions [14].
(TIF)

S10 Fig Correlation analysis on growth parameters for different groups of yeasts. Several growth kinetic parameters were investigated for their correlations to energy metabolism and growth in different metabolic groups of yeasts. The groups consist of purely respiring (blue), pre-WGD respiro-fermenting (green), WGD respiro-fermenting (orange) and yeast with glucose repressed respiration (red). Correlation coefficients (Spearman’s rho) between parameters are shown in the upper panels, histograms of data distribution are shown in the diagonal panels, and scatterplot with linear trend lines are shown in the lower panels. **(A)** This figure confirms that respiration is the most energy efficient producing pathway in all yeasts, which is indicated by high and positive correlation between respiration (O_2_ consumption and CO_2_ production rates) and ATP production rates. **(B)** Respiring yeasts do not exhibit overflow metabolism, they are energy efficient and their growth and glucose uptake is highly coupled to ATP production rates. This is indicated by high and positive correlation between growth (DW production rates), respiration, glucose consumption rates and ATP production rates. **(C)** Yeasts that exhibit “overconsumption” of glucose, which is expressed as overflow metabolism that enable high ATP production rates, are less dependent on respiration for cell-proliferation as compared to respiring yeasts. This is further indicated by high and positive correlation between parameters such as respiration and fermentation (CO_2_ and ethanol production rates) with ATP production rates, of which growth is less correlated with as compared to respiring yeasts. **(D)** The original trait of Crabtree effect is equivalent to glucose repression of respiration in favor of increased ethanol fermentation. In other words, a trade-off between respiration and fermentation should be observed among these yeasts. This could in theory result in reduced ATP production and increased dependency on fermentation for energy production and growth. Our data reveal that glucose repression of respiration does occur, as a settled trait, in at least *S. cerevisiae* and closest related species that belong to the *Saccharomyces* and *Kazachstania* clades. This is indicated by a higher positive correlation between fermentation and growth that results in reduced ATP production rates among these yeasts.
(TIF)

S1 Script Statistical tests and analysis. (ZIP)

S1 Table Yeast long-term Crabtree effect — Growth kinetics for all experiments. (PDF)

S2 Table Statistical tests on growth rates among metabolic groups. (PDF)

## References

[1] De Deken RH (1966) The Crabtree effect: a regulatory system in yeast. J Gen Microbiol 44: 149–156. 10.1099/00221287-44-2-149 5969497

[2] Postma E, Verduyn C, Scheffers WA, Van Dijken JP (1989) Enzymic analysis of the crabtree effect in glucose-limited chemostat cultures of *Saccharomyces cerevisiae* . Appl Environ Microbiol 55: 468–477. 2566299 10.1128/aem.55.2.468-477.1989 PMC184133

[3] Westergaard SL, Oliveira AP, Bro C, Olsson L, Nielsen J (2007) A systems biology approach to study glucose repression in the yeast *Saccharomyces cerevisiae* . Biotechnol Bioeng 96: 134–145. 10.1002/bit.21135 16878332

[4] Kim JH, Roy A, Jouandot D 2nd, Cho KH (2013) The glucose signaling network in yeast. Biochim Biophys Acta 1830: 5204–5210. 10.1016/j.bbagen.2013.07.025 23911748 PMC3785329

[5] Conrad M, Schothorst J, Kankipati HN, Van Zeebroeck G, Rubio-Texeira M, et al (2014) Nutrient sensing and signaling in the yeast *Saccharomyces cerevisiae* . FEMS Microbiol Rev. 10.1111/1574-6976.12065 24483210 PMC4238866

[6] Broach JR (2012) Nutritional Control of Growth and Development in Yeast. Genetics 192: 73–105. 10.1534/genetics.111.135731 22964838 PMC3430547

[7] Kaspar von Meyenburg H (1969) Energetics of the budding cycle of *Saccharomyces cerevisiae* during glucose limited aerobic growth. Arch Mikrobiol 66: 289–303. 10.1007/BF00414585 5384632

[8] Barford J, Hall R (1981) A mathematical model for the aerobic growth of *Saccharomyces cerevisiae* with a saturated respiratory capacity. Biotechnol Bioeng 23: 1735–1762. 10.1002/bit.260230806

[9] Sonnleitner B, Kappeli O (1986) Growth of *Saccharomyces cerevisiae* is controlled by its limited respiratory capacity: Formulation and verification of a hypothesis. Biotechnol Bioeng 28: 927–937. 10.1002/bit.260280620 18555411

[10] Alexander MA, Jeffries TW (1990) Respiratory Efficiency and Metabolite Partitioning as Regulatory Phenomena in Yeasts. Enzyme Microb Technol 12: 2–19. 10.1016/0141-0229(90)90173-N

[11] Rieger M, Kappeli O, Fiechter A (1983) The Role of Limited Respiration in the Incomplete Oxidation of Glucose by *Saccharomyces-Cerevisiae* . J Gen Microbiol 129: 653–661.

[12] Pronk JT, Yde Steensma H, Van Dijken JP (1996) Pyruvate metabolism in *Saccharomyces cerevisiae* . Yeast 12: 1607–1633. 10.1002/(SICI)1097-0061(199612)12:16<1607::AID-YEA70>3.0.CO;2-4 9123965

[13] Petrik M, Kappeli O, Fiechter A (1983) An Expanded Concept for the Glucose Effect in the Yeast *Saccharomyces-Uvarum* — Involvement of Short-Term and Long-Term Regulation. J Gen Microbiol 129: 43–49.

[14] Piskur J, Rozpedowska E, Polakova S, Merico A, Compagno C (2006) How did *Saccharomyces* evolve to become a good brewer? Trends Genet 22: 183–186. 10.1016/j.tig.2006.02.002 16499989

[15] Merico A, Sulo P, Piskur J, Compagno C (2007) Fermentative lifestyle in yeasts belonging to the *Saccharomyces* complex. The FEBS journal 274: 976–989. 10.1111/j.1742-4658.2007.05645.x 17239085

[16] Hagman A, Sall T, Compagno C, Piskur J (2013) Yeast “make-accumulate-consume” life strategy evolved as a multi-step process that predates the whole genome duplication. PLoS One 8: e68734 10.1371/journal.pone.0068734 23869229 PMC3711898

[17] Hagman A, Sall T, Piskur J (2014) Analysis on yeast short-term Crabtree effect and its origin. FEBS J. 10.1111/febs.13019 25161062 PMC4240471

[18] Gojkovic Z, Knecht W, Zameitat E, Warneboldt J, Coutelis JB, et al (2004) Horizontal gene transfer promoted evolution of the ability to propagate under anaerobic conditions in yeasts. Molecular Genetics and Genomics: MGG 271: 387–393. 10.1007/s00438-004-0995-7 15014982

[19] Moller K, Olsson L, Piskur J (2001) Ability for anaerobic growth is not sufficient for development of the petite phenotype in *Saccharomyces kluyveri* . J Bacteriol 183: 2485–2489. 10.1128/JB.183.8.2485-2489.2001 11274107 PMC95164

[20] Dujon B (2010) Yeast evolutionary genomics. Nature reviews Genetics 11: 512–524. 10.1038/nrg2811 20559329

[21] Marcet-Houben M, Marceddu G, Gabaldon T (2009) Phylogenomics of the oxidative phosphorylation in fungi reveals extensive gene duplication followed by functional divergence. BMC Evol Biol 9: 295 10.1186/1471-2148-9-295 20025735 PMC2803194

[22] Huberts DH, Niebel B, Heinemann M (2012) A flux-sensing mechanism could regulate the switch between respiration and fermentation. FEMS Yeast Res 12: 118–128. 10.1111/j.1567-1364.2011.00767.x 22129078

[23] Chen XJ, Wesolowski-Louvel M, Fukuhara H (1992) Glucose transport in the yeast *Kluyveromyces lactis*. II. Transcriptional regulation of the glucose transporter gene *RAG1* . Mol Gen Genet 233: 97–105. 10.1007/BF00587566 1603079

[24] Billard P, Menart S, Blaisonneau J, BolotinFukuhara M, Fukuhara H, et al (1996) Glucose uptake in *Kluyveromyces lactis*: Role of the *HGT1* gene in glucose transport. J Bacteriol 178: 5860–5866. 8830679 10.1128/jb.178.20.5860-5866.1996 PMC178439

[25] van Urk H, Postma E, Scheffers WA, van Dijken JP (1989) Glucose transport in crabtree-positive and crabtree-negative yeasts. J Gen Microbiol 135: 2399–2406. 2628542 10.1099/00221287-135-9-2399

[26] Lin Z, Li WH (2011) Expansion of hexose transporter genes was associated with the evolution of aerobic fermentation in yeasts. Mol Biol Evol 28: 131–142. 10.1093/molbev/msq184 20660490 PMC3002240

[27] Ihmels J, Bergmann S, Gerami-Nejad M, Yanai I, McClellan M, et al (2005) Rewiring of the yeast transcriptional network through the evolution of motif usage. Science 309: 938–940. 10.1126/science.1113833 16081737

[28] Conant GC, Wolfe KH (2007) Increased glycolytic flux as an outcome of whole-genome duplication in yeast. Molecular Systems Biology 3: 129 10.1038/msb4100170 17667951 PMC1943425

[29] Kurtzman CP, Robnett CJ (2003) Phylogenetic relationships among yeasts of the ‘*Saccharomyces* complex’ determined from multigene sequence analyses. FEMS Yeast Res 3: 417–432. 10.1016/S1567-1356(03)00012-6 12748053

[30] Wolfe KH, Shields DC (1997) Molecular evidence for an ancient duplication of the entire yeast genome. Nature 387: 708–713. 10.1038/43168 9192896

