# Supplementary figures and images for: A Study on the Fundamental Mechanism and the Evolutionary Driving Forces behind Aerobic Fermentation in Yeast
Source: PLoS One. 2015 Jan 24;10(1):e0116942. doi: 10.1371/journal.pone.0116942 (PMC4305316; doi:10.1371/journal.pone.0116942)

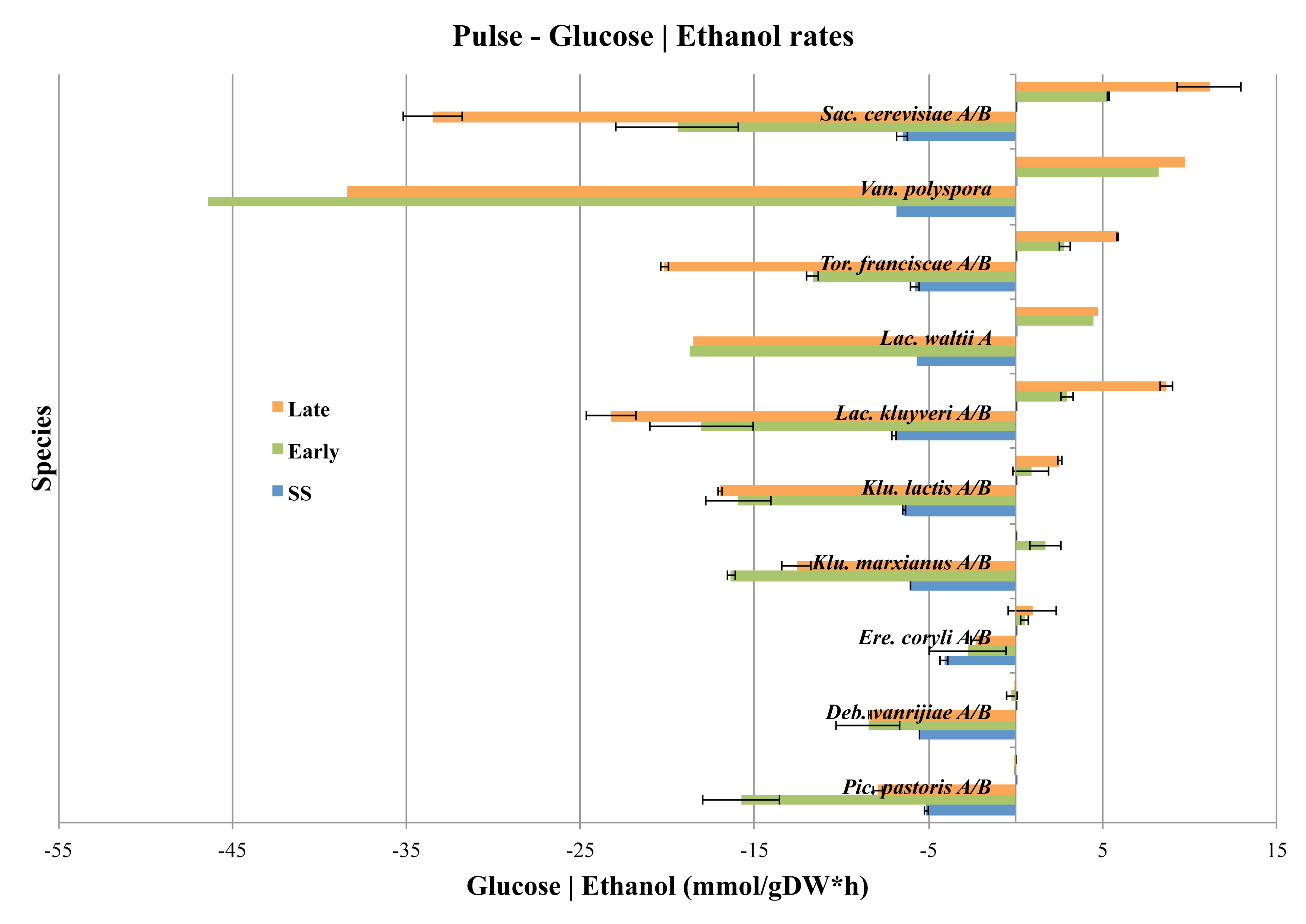

Supplement: S1 Fig — All short-term Crabtree positive yeasts already possess an upregulated anaerobic glycolytic pathway under aerobic, glucose limited, and fully respiring steady-state growth at low rates (SS). This was expressed as an immediate ethanol formation within 20 minutes (Early-phase) after a glucose pulse in all the fermenting yeasts. Several of the fermenting yeast species such as, S. cerevisiae, T. franciscae and L. kluyverii also significantly increased their glucose uptake rates, and ethanol production rates at time intervals later than 60 minutes (Late-phase) after a glucose pulse. The average glucose uptake and ethanol production rates from two biological replicates with error bars corresponding to the standard deviation are illustrated. (TIF) [file pone.0116942.s001.tif]

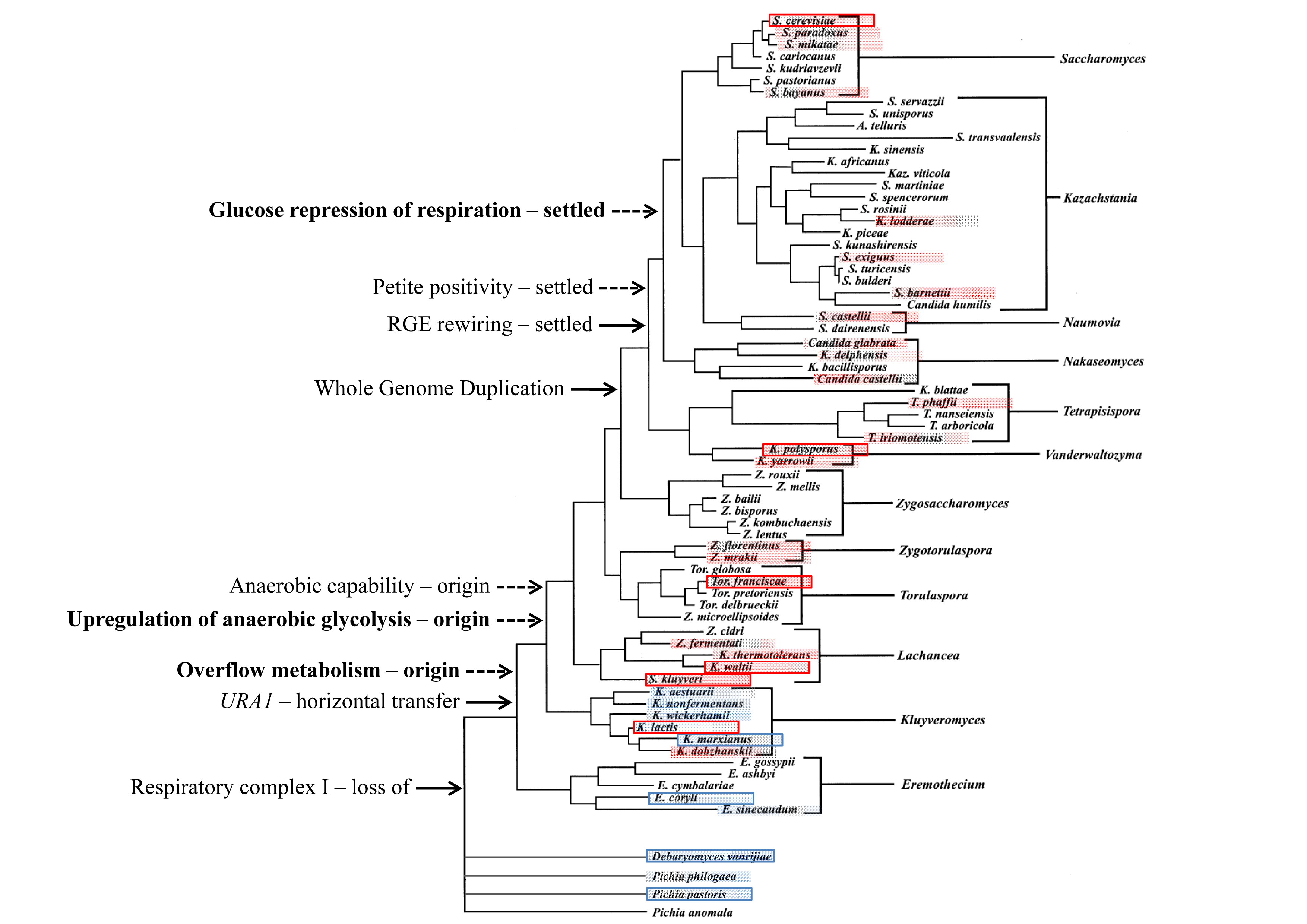

Supplement: S2 Fig — This figure illustrate the distribution of short-term Crabtree effect and long-term Crabtree effect in all the investigated yeasts from our previous studies [16, 17]. Yeast species that are short-term Crabtree positive (framed in red), short-term Crabtree negative (framed in blue), long-term Crabtree positive (highlighted in red), and long-term Crabtree negative (highlighted in blue) are shown. Several evolutionary events that are relevant for the modern traits are also shown. Some events that have left a clear fingerprint in the modern genomes (whole arrows), such as the rewiring of RGE (rapid growth elements) [27], the whole genome duplication event [30], the horizontal transfer of URA1 [18], and the loss of respiratory complex I [20], have been more precisely timed, while the timing of complex traits (broken arrows), such as overflow metabolism, long-term upregulation of anaerobic glycolysis and glucose repression of respiration (in bold), petite positivity and the capability of anaerobic growth [15], might be less precise. Figure adapted from [29]. (TIF) [file pone.0116942.s002.tif]

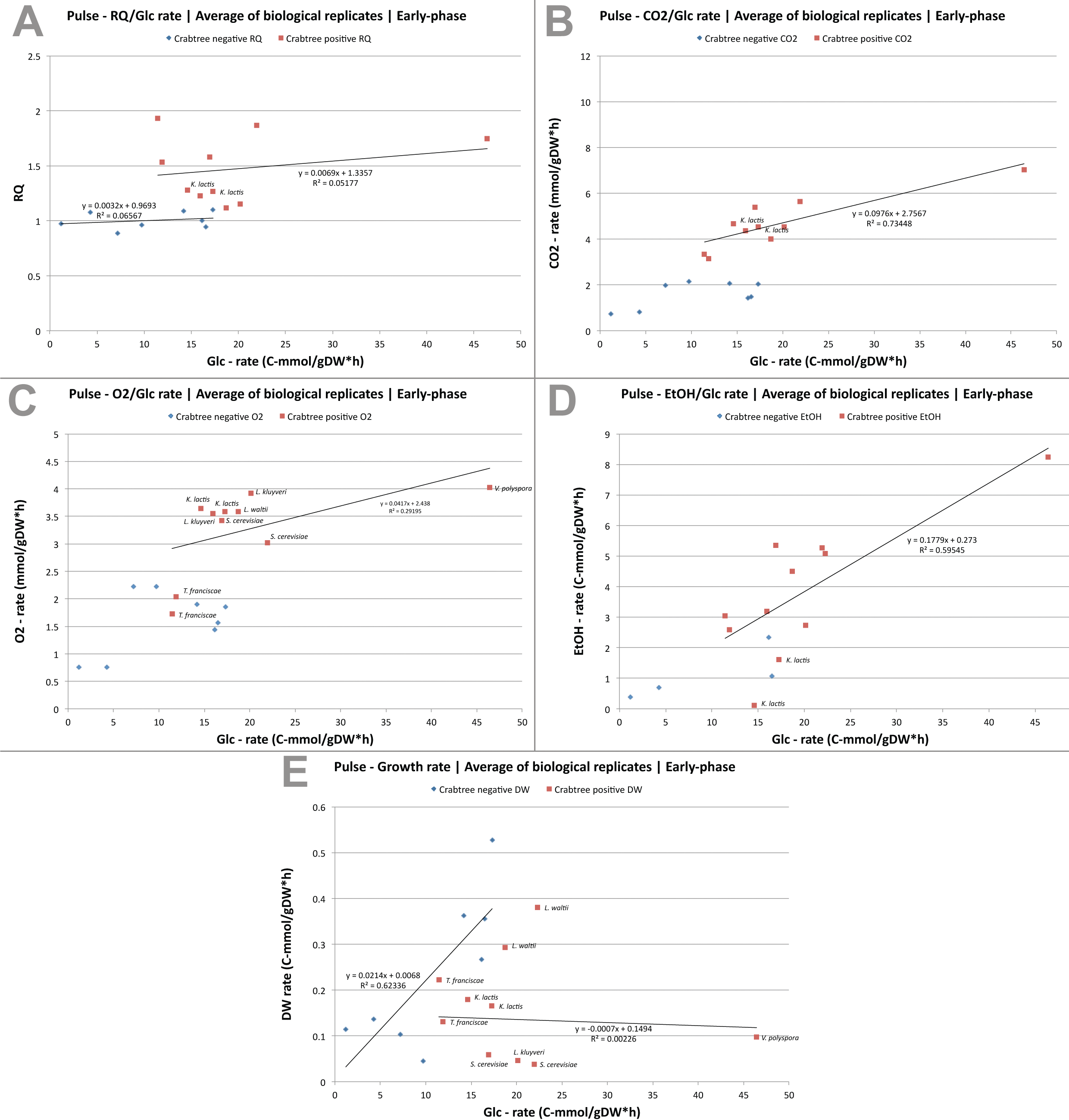

Supplement: S3 Fig — Similar analysis as in Fig. 7, but only for early time intervals, up to 20 minutes after a glucose-pulse. The data is highly variable due to low and unstable growth, and cultures had yet to adapt to the new glucose-rich environment (see also S4 Fig.). (TIF) [file pone.0116942.s003.tif]

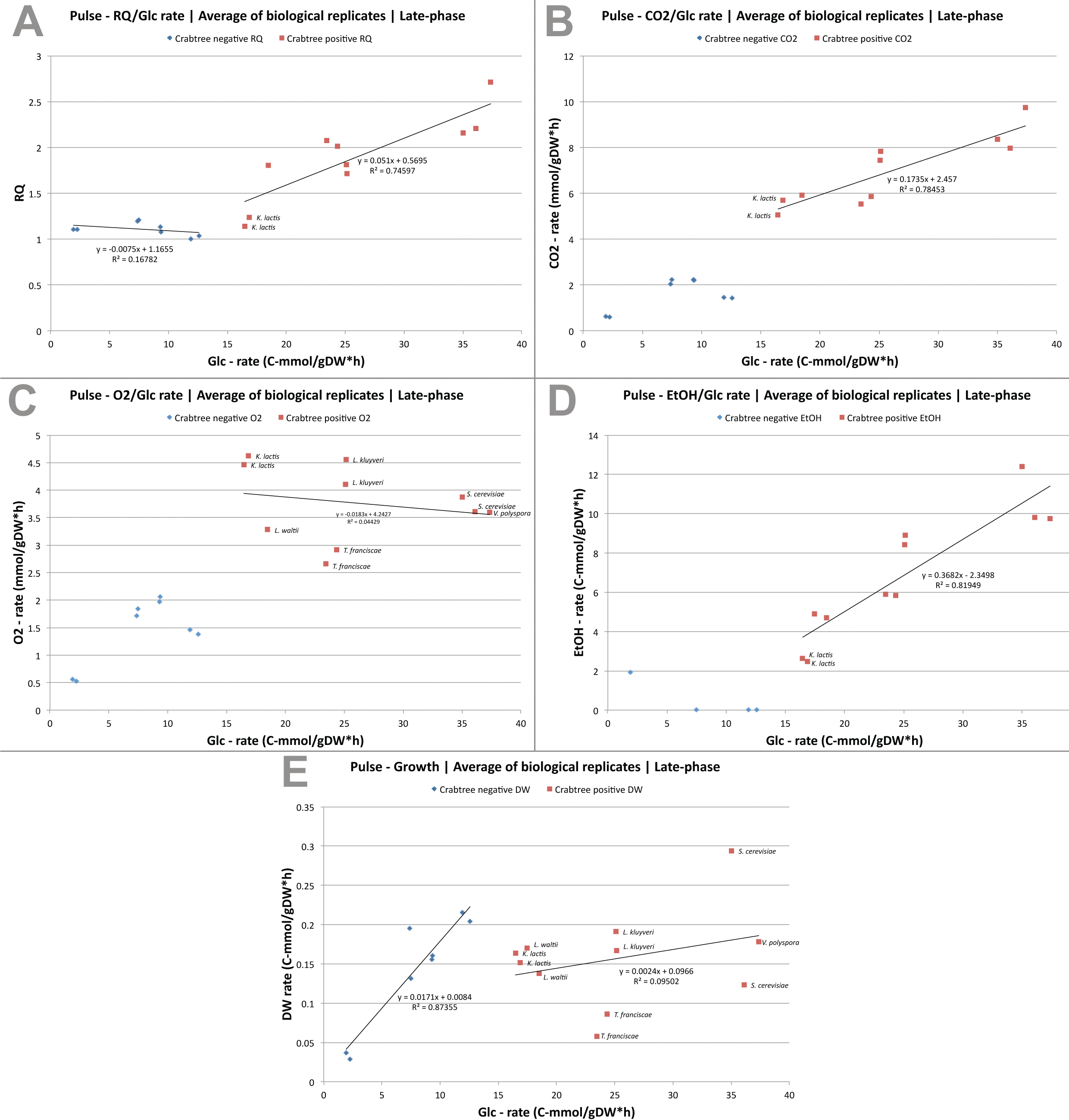

Supplement: S4 Fig — This figure illustrate a similar analysis as in Fig. 7, but only for time intervals later than 60 minutes after a glucose-pulse, when the cultures were more adapted to the new growth conditions. The data is less variable as compared to earlier time intervals (S3 Fig.). (TIF) [file pone.0116942.s004.tif]

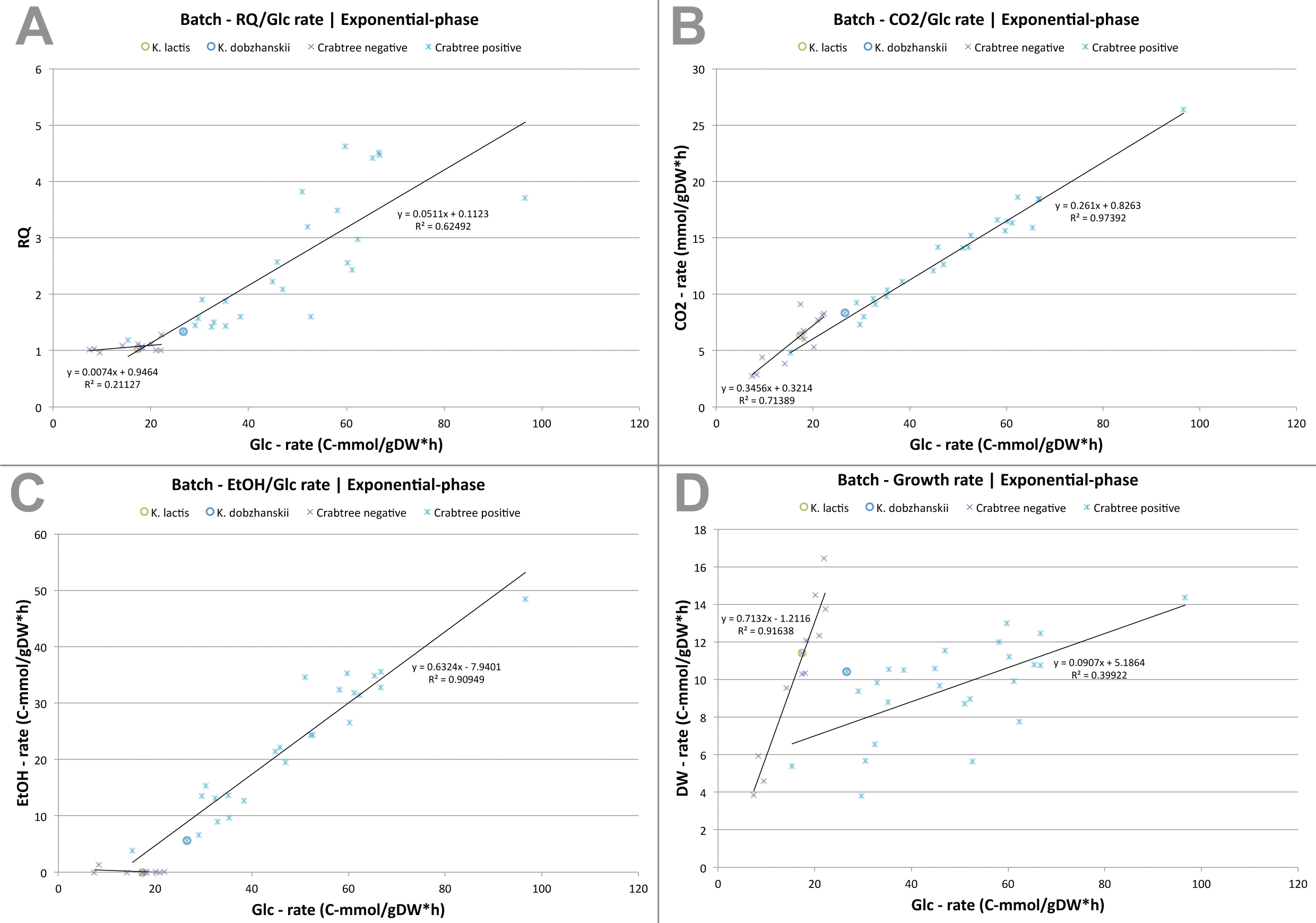

Supplement: S5 Fig — Similar analysis as in Fig. 7, but the data is from a study of forty different yeast species from batch cultures [16]. Under these conditions, the cultures are fully adapted to high glucose levels and are growing at their specific growth rates. The dataset is highly correlated and it is therefore possible to determine more precisely the average GFcrit among species to 15 C-mmole/gDW*h (see Materials and Methods section for further information). (TIF) [file pone.0116942.s005.tif]

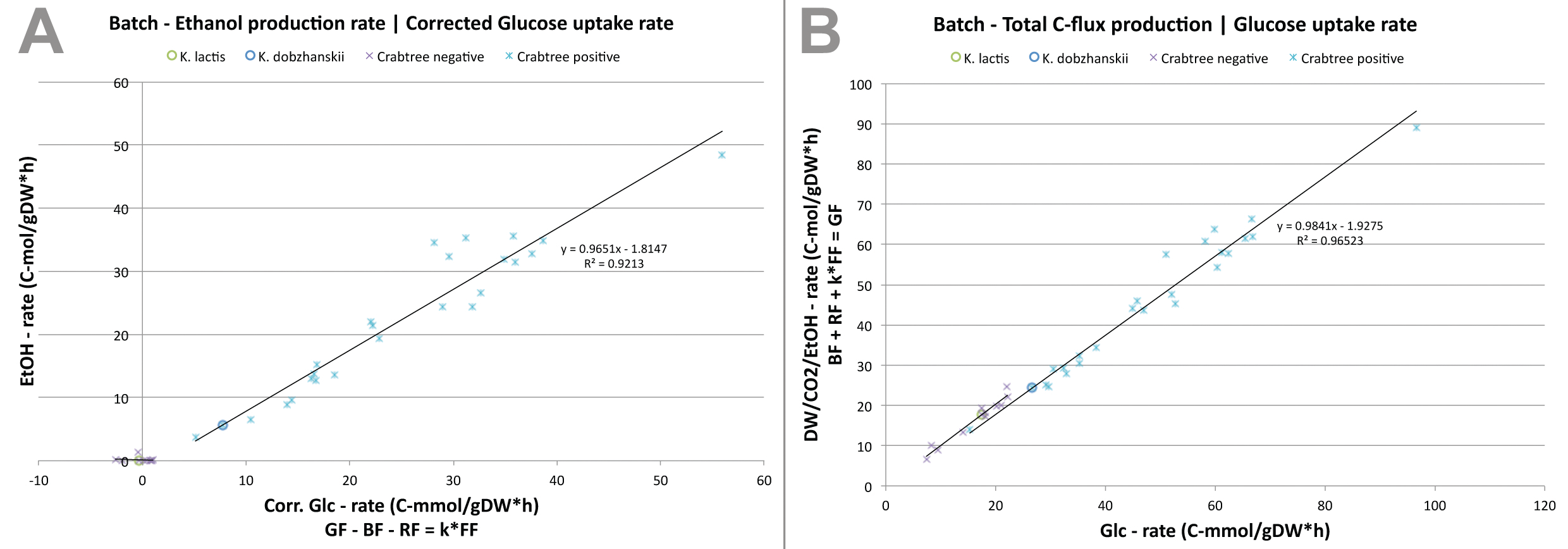

Supplement: S6 Fig — When glucose uptake rates exceed the sum of carbon flow through biomass formation and respiration, an overflow through anaerobic glycolysis is observed, which is predicted by model (1). (A) An interrelationship between evolutionary conserved metabolic pathways, which occurs in a linear fashion that spans at least the evolutionary history of over forty investigated yeast species (and perhaps as far back as to the formation of dikarya-clade or even further). The slope (k), which can be derived from the plot equals 1, and correspond to the relationship between fermentative flux (FF) and glycolytic flux (GF) above the critical glucose uptake rate (GFcrit), according to model (1). (B) C-flux balancing was used to validate model (1), which is illustrated in this figure. (TIF) [file pone.0116942.s006.tif]

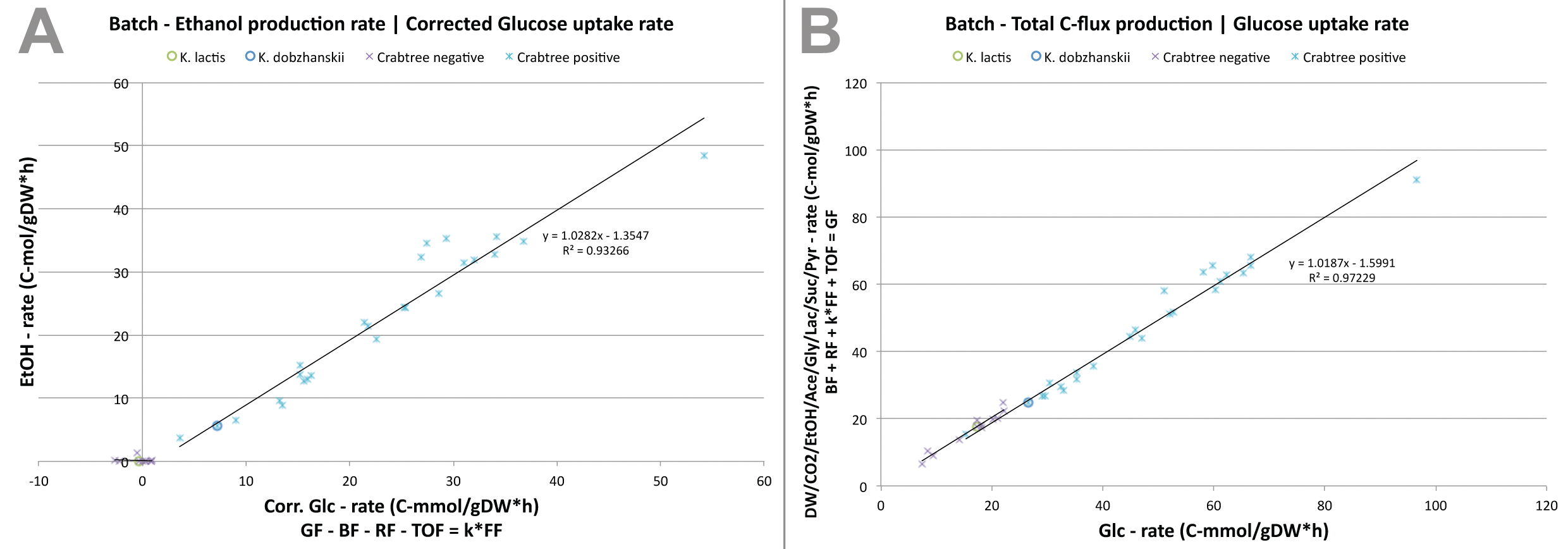

Supplement: S7 Fig — Similar analysis was made as illustrated in S6A and B Fig., but with an additional variable in model (2), in an attempt to improve the model. Some variation in the dataset is caused by the formation of other overflow products, which is not accounted for in model (1). This model is already highly linearly correlated with an R2 value of 96, so the incorporation of an extra variable only improves the model slightly to an R2 value of 97 (compare S7B Fig. with S6B Fig.). (TIF) [file pone.0116942.s007.tif]

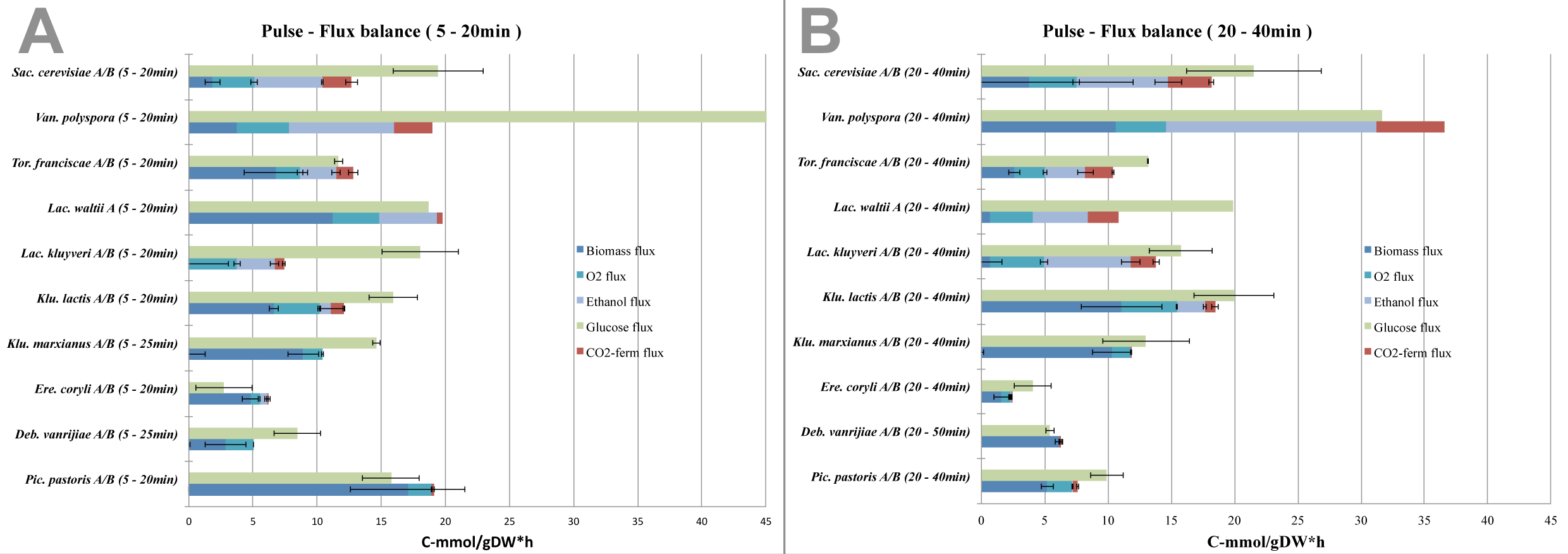

Supplement: S8 Fig — This figures illustrate a carbon-flux balance similar to Fig. 4, but for the time intervals (A) 5 to 20 minutes and (B) 20 to 40 minutes. Apart from the observations and conclusions that can be drawn in Fig. 4, this figure further illustrates the unstable growth that sets in upon a sudden release from glucose limiting growth. The average of two biological replicates with the corresponding standard deviation is illustrated. Data was obtained from [17]. (TIF) [file pone.0116942.s008.tif]

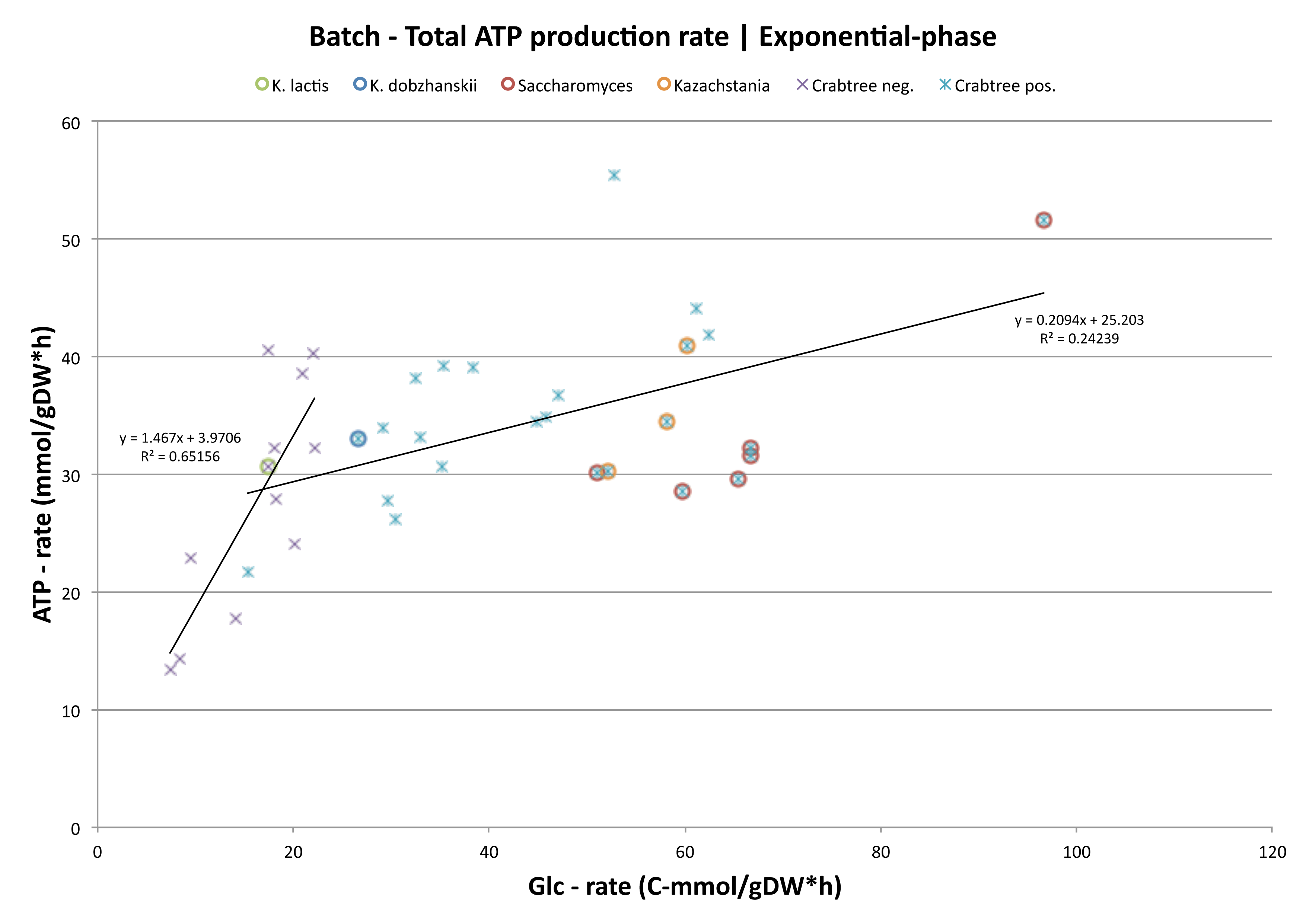

Supplement: S9 Fig — When the sum of theoretical ATP production rates from anaerobic glycolysis and respiration are plotted against glucose consumption rates, a general trend can be observed. Increased glucose consumption rates can result in higher ATP production rates. Hence, overflow metabolism enables increased ATP production rates for cell-proliferation on glucose. This figure also shows that S. cerevisiae and a majority of its closely related species, in the Saccharomyces and Kazachstania clades, appear to lack increased ATP production rates, despite high glycolytic flux. This can be explained by glucose repression of respiration, which is a trait that appears to have evolved late in modern yeasts. Glucose repression of respiration could have provided other competitive advantages in glucose rich conditions [14]. (TIF) [file pone.0116942.s009.tif]

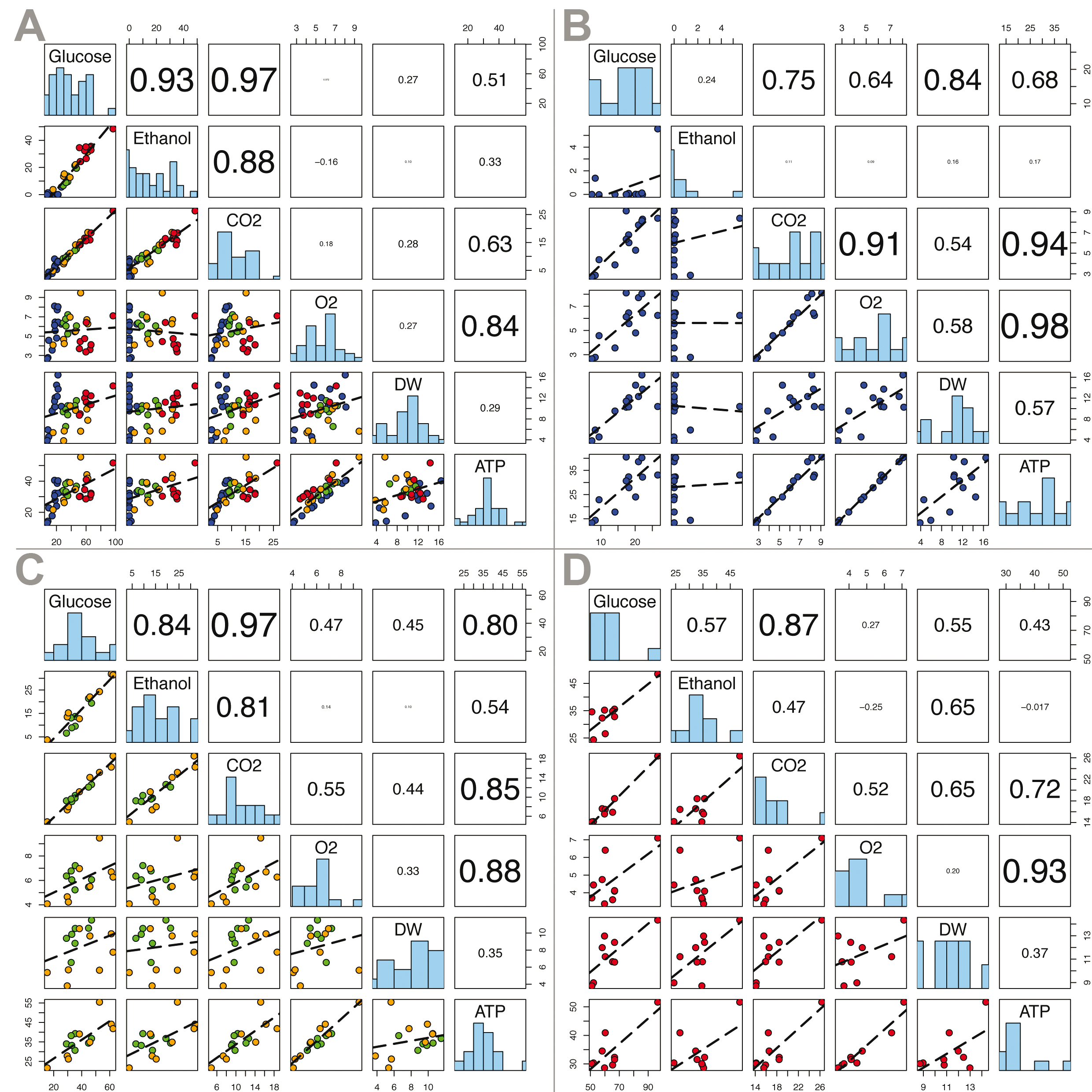

Supplement: S10 Fig — Several growth kinetic parameters were investigated for their correlations to energy metabolism and growth in different metabolic groups of yeasts. The groups consist of purely respiring (blue), pre-WGD respiro-fermenting (green), WGD respiro-fermenting (orange) and yeast with glucose repressed respiration (red). Correlation coefficients (Spearman’s rho) between parameters are shown in the upper panels, histograms of data distribution are shown in the diagonal panels, and scatterplot with linear trend lines are shown in the lower panels. (A) This figure confirms that respiration is the most energy efficient producing pathway in all yeasts, which is indicated by high and positive correlation between respiration (O2 consumption and CO2 production rates) and ATP production rates. (B) Respiring yeasts do not exhibit overflow metabolism, they are energy efficient and their growth and glucose uptake is highly coupled to ATP production rates. This is indicated by high and positive correlation between growth (DW production rates), respiration, glucose consumption rates and ATP production rates. (C) Yeasts that exhibit “overconsumption” of glucose, which is expressed as overflow metabolism that enable high ATP production rates, are less dependent on respiration for cell-proliferation as compared to respiring yeasts. This is further indicated by high and positive correlation between parameters such as respiration and fermentation (CO2 and ethanol production rates) with ATP production rates, of which growth is less correlated with as compared to respiring yeasts. (D) The original trait of Crabtree effect is equivalent to glucose repression of respiration in favor of increased ethanol fermentation. In other words, a trade-off between respiration and fermentation should be observed among these yeasts. This could in theory result in reduced ATP production and increased dependency on fermentation for energy production and growth. Our data reveal that glucose repression of respirat [file pone.0116942.s010.tif]
