# Supplementary material for: A Study on the Fundamental Mechanism and the Evolutionary Driving Forces behind Aerobic Fermentation in Yeast
Source: PLoS One. 2015 Jan 24;10(1):e0116942. doi: 10.1371/journal.pone.0116942 (PMC4305316; doi:10.1371/journal.pone.0116942)
Supplement: S1 Table — (PDF) [file pone.0116942.s012.pdf]

Table S1. Yeast long-term Crabtree effect – Growth kinetics for all experiments.

Species	Y	Cons. rate*: Glucose	Prod. rate*: Ethanol	Prod. rate*: Glycerol	Prod. rate*: Acetate	Prod. rate*: Pyruvate	Prod. rate*: Succinate	Prod. rate*: Lactate	Growth rate*: DW	Prod. rate*: CO2	Cons. Rate**: O2	RQ
Sac. p Weihenstephan A2	Y1288	47,97	32,59	0,62	0,00	0,08	0,00	0,00	8,90	12,93	3,21	4,03
Sac. p Weihenstephan A1	Y1288	54,05	36,62	0,64	0,00	0,11	0,00	0,00	8,53	15,33	4,21	3,65
Sac. cerevisiae A2	Y706	72,32	34,97	2,19	0,99	0,22	0,00	0,23	11,10	18,92	4,20	4,51
Sac. cerevisiae A1	Y706	61,14	30,71	2,00	1,10	0,22	0,00	0,17	10,40	17,93	4,04	4,43
Sac. paradoxus	Y052	59,77	35,24	1,73	0,00	0,15	0,00	0,00	12,98	15,59	3,37	4,63
Sac. mikatae	Y393	96,60	48,49	1,43	0,00	0,32	0,00	0,00	14,34	26,33	7,10	3,71
Sac. uvarum	Y1124	65,37	34,82	1,16	0,00	0,43	0,00	0,36	10,78	15,91	3,60	4,42
Sac. eubayanus	Y1693	66,69	35,59	1,38	0,00	0,29	0,00	0,00	12,44	18,44	4,09	4,51
Kaz. lodderae A2	Y489	62,23	27,08	1,93	0,99	0,38	0,91	0,00	11,64	16,87	6,60	2,55
Kaz. lodderae A1	Y489	58,31	26,09	1,99	0,74	0,37	0,84	0,00	10,78	15,99	6,22	2,57
Kaz. exiguus	Y670	58,16	32,34	0,71	0,26	0,26	1,00	0,46	11,99	16,57	4,75	3,49
Kaz. barnettii	Y477	52,11	24,36	2,40	0,54	0,40	0,00	0,30	8,98	14,17	4,44	3,19
Nau. castellii A1	Y056	52,70	24,35	4,27	0,81	0,35	0,81	0,24	5,64	15,19	9,47	1,60
Nak. glabrata A2	Y475	31,74	16,24	1,65	0,00	0,17	0,00	0,00	5,83	8,17	4,69	1,74
Nak. glabrata A1	Y475	29,21	14,31	1,19	0,00	0,17	0,00	0,00	5,55	7,83	3,76	2,08
Nak. delphensis	476	62,38	31,45	4,60	0,00	0,35	0,00	0,00	7,76	18,62	6,27	2,97
Nak. castellii A2	Y484	15,32	3,75	1,16	0,00	0,06	0,31	0,00	5,37	4,84	4,08	1,19
Tet. phaffii	Y482	29,64	13,53	2,11	0,00	0,07	0,00	0,00	3,81	7,34	4,66	1,57
Tet. iriomotensis A2	Y1299	36,43	11,29	0,90	0,00	0,08	0,00	0,00	10,15	10,85	7,00	1,55
Tet. iriomotensis A1	Y1299	40,25	14,20	1,24	0,00	0,11	0,00	0,00	10,88	11,41	6,93	1,65
Van. polysporus A1	Y1293	61,13	31,88	1,87	0,00	0,41	0,61	0,00	9,91	16,30	6,70	2,43
Van. yarrowii	1677	45,84	22,07	0,50	0,00	0,11	0,00	0,00	9,66	14,14	5,51	2,57
Zto. florentinus A2	Y479	44,82	22,08	0,16	0,00	0,24	0,00	0,00	10,58	12,46	5,45	2,28
Zto. florentinus A1	Y479	44,92	20,76	0,16	0,00	0,25	0,00	0,00	10,57	11,77	5,46	2,15
Zto. mrakii	Y480	32,45	13,08	0,10	0,00	0,26	0,00	0,00	6,55	9,62	6,78	1,42
Tor. franciscae A2	Y1055	31,61	8,76	0,19	0,00	0,33	0,00	0,00	9,97	8,94	6,01	1,49
Tor. franciscae A1	Y1055	34,17	9,03	0,14	0,00	0,33	0,00	0,00	9,74	9,21	6,09	1,51
Lac. fermentati	Y083	47,01	19,42	0,07	0,00	0,24	0,00	0,00	11,52	12,65	6,06	2,09
Lac. thermotolerans	Y688	35,20	13,68	0,25	0,91	0,21	0,00	0,00	8,80	9,81	5,23	1,88
Lac. waltii A2	Y1062	29,08	6,58	0,14	0,36	0,94	0,00	0,00	9,38	9,24	6,36	1,45
Lac. kluyverii A2	Y057	36,35	10,25	0,33	0,43	0,28	0,22	0,00	10,68	10,51	7,21	1,46
Lac. kluyverii A1	Y057	34,34	8,89	0,29	0,47	0,25	0,20	0,00	10,40	10,23	7,22	1,42
Klu. aestuarii	Y797	21,97	0,12	0,03	0,00	0,01	0,00	0,00	16,45	8,13	8,04	1,01
Klu. nonfermentans	Y1057	7,40	0,00	-0,02	0,00	-0,01	0,01	0,00	3,86	2,73	2,67	1,02
Klu. wickerhamii	Y113	20,97	0,07	0,06	0,00	0,02	0,00	0,00	12,33	7,72	7,72	1,00
Klu. lactis A2	Y707	15,89	0,01	0,00	0,00	-0,01	0,00	0,00	9,79	5,70	5,72	1,00
Klu. lactis A1	Y707	18,92	-0,06	-0,14	0,00	0,01	0,00	0,00	13,07	6,99	6,54	1,07

Species	Y	Cons. rate*: Glucose	Prod. rate*: Ethanol	Prod. rate*: Glycerol	Prod. rate*: Acetate	Prod. rate*: Pyruvate	Prod. rate*: Succinate	Prod. rate*: Lactate	Growth rate*: DW	Prod. rate*: CO2	Cons. Rate**: O2	RQ
Klu. marxianus C	Y1674	22,16	-0,02	0,27	0,00	0,02	0,00	0,00	13,74	8,28	6,44	1,29
Klu. marxianus B	Y1675	18,10	0,09	0,26	-0,15	0,00	0,00	0,00	10,34	6,77	6,46	1,05
Klu. marxianus A2	Y1058	18,29	0,01	0,06	0,00	0,00	0,00	0,00	12,13	5,95	5,46	1,09
Klu. marxianus A1	Y1058	18,10	0,00	0,08	0,00	0,00	0,00	0,00	12,06	6,07	5,68	1,07
Klu. dobzhanskii A1	Y796	26,58	5,56	0,46	0,00	0,11	0,00	0,00	10,41	8,38	6,25	1,34
Ere. coryli A2	Y999	8,45	1,49	0,00	0,00	0,10	0,00	0,00	6,06	2,79	2,64	1,06
Ere. coryli A1	Y999	8,30	1,26	0,00	0,00	0,10	0,00	0,00	5,78	2,95	2,93	1,01
Ere. sinecaudum A2	Y1002	9,32	-0,01	0,00	0,00	0,10	0,00	0,00	4,49	4,30	4,52	0,95
Ere. sinecaudum A1	Y1002	9,66	-0,03	-0,01	0,00	0,10	0,00	0,00	4,68	4,46	4,63	0,96
Deb. vanrijiae A2	Y060	21,39	0,00	0,14	0,00	0,00	0,00	0,00	15,69	5,53	4,84	1,14
Deb. vanrijiae A1	Y060	18,88	0,02	0,05	0,00	0,00	0,00	0,00	13,31	4,61	4,19	1,10
Pic. philogaea	Y074	14,13	0,02	0,03	0,20	0,11	0,00	0,00	9,55	3,87	4,37	0,88
Pic. pastoris	Y1294	17,40	0,96	0,01	0,00	0,11	0,12	0,00	10,29	9,09	8,11	1,12

* C-mmol/gDW*h
** mmol/gDW*h
Data extracted from Hagman et al. PLOS one 2013
