# Supplementary material for: A Study on the Fundamental Mechanism and the Evolutionary Driving Forces behind Aerobic Fermentation in Yeast
Source: PLoS One. 2015 Jan 24;10(1):e0116942. doi: 10.1371/journal.pone.0116942 (PMC4305316; doi:10.1371/journal.pone.0116942)
Supplement: S2 Table — (PDF) [file pone.0116942.s013.pdf]

Table S2. Statististical tests on growth rates among metabolic groups.

Parameter X	Parameter Y	Statistical analysis2	α (5%) sign.	p-value	df
Group 1	Group 2	Welch Two Sample t-test	No	5,06E-01	17,5
		Wilcoxon rank sum test	No	4,38E-01	
Group 2	Group 3	Welch Two Sample t-test	No	8,68E-01	19,9
		Wilcoxon rank sum test	No	9,51E-01	
Group 1	Group 3	Welch Two Sample t-test	No	4,48E-01	22,3
		Wilcoxon rank sum test	No	4,08E-01	

Group 1 - Purely respiring yeasts

Group 2 - Respiro-fermenting yeasts excluding *Kazachstania*/*Saccharomyces* yeasts

Group 3 - *Kazachstania*/*Saccharomyces* yeasts
